# Toosendanin Alleviates Cerebral Ischemia/Reperfusion Injury via Inhibiting Neural Ferroptosis Through Lipid Metabolic Reprogramming in MCAO Mice

**DOI:** 10.1002/cns.70952

**Published:** 2026-05-29

**Authors:** Xinyun Li, Zhiyong Zhao, Jingting Zhao, Xiangming Ye, Zhenfei Xiong, Jiejin Zhao

**Affiliations:** ^1^ School of Rehabilitation Hangzhou Medical College Hangzhou China; ^2^ Children's Hospital, Zhejiang University School of Medicine National Clinical Research Center for Child Health Hangzhou China; ^3^ Department of Rehabilitation Medicine Zhejiang Provincial People's Hospital, Affiliated People's Hospital, Hangzhou Medical College Hangzhou China; ^4^ Department of Foot and Ankle, Department of Foot and Ankle Surgery Xiaoshan District Hospital of Traditional Chinese Medicine Hangzhou China; ^5^ Department of Rehabilitation Medicine The First People's Hospital of Xiaoshan District Hangzhou China

**Keywords:** ACSL4, cerebral ischemia/reperfusion injury, ferroptosis, lipid metabolic reprogramming (LMR), Toosendanin

## Abstract

**Background:**

The crosstalk between ferroptosis and neuroinflammation plays an important role in the pathogenesis of cerebral ischemia–reperfusion injury. Toosendanin (TSN), a triterpenoid compound, exhibits a wide range of pharmacological activities in human diseases. Here we investigated the potential neuroprotective effects of TSN in cerebral ischemia–reperfusion injury.

**Methods:**

In our study, in vivo murine middle cerebral artery occlusion (MCAO) model and in vitro oxygen–glucose deprivation/reoxygenation (OGD/R) model were constructed to mimic cerebral ischemia–reperfusion injury. TTC staining, open‐field test, Morris water maze test, hanging wire test, rotarod test, foot‐fault test, hematoxylin and eosin (H&E) staining, flow cytometry, western blot and reverse transcription‐quantitative PCR were conducted to evaluate the potential influence of TSN in cerebral ischemia–reperfusion injury.

**Results:**

Our results indicated that post‐stroke administration of TSN significantly reduced infarct volume and improved long‐term functional recovery of MCAO mice. Mechanistically, TSN alleviated neural oxidative stress, lipid peroxidation, and ferroptosis in MCAO mice or in vitro after OGD/R. In addition, TSN modulated the infiltrating of immune cells in MCAO mice in vivo and T cell differentiation in vitro. Mechanistically, TSN directly interacted with ACSL4 and suppressed its enzymatic activity, thus suppressing the ACSL4/LPCAT3 axis to reduce the incorporation of pro‐ferroptotic polyunsaturated fatty acids (PUFA) into phospholipids, and finally inhibiting lipid peroxidation and neural ferroptosis. In addition, the induction of ferroptosis abrogated the influence of TSN on immune infiltration and lipid metabolism in MCAO mice.

**Conclusion:**

Our findings identify TSN as a promising therapeutic agent for cerebral ischemia–reperfusion injury via inhibiting ACSL4‐mediated ferroptosis.

## Graphical abstract

Post‐stroke administration of TSN significantly reduced infarct volume, neuroinflammation, and improved functional recovery of MCAO mice. TSN alleviated neural oxidative stress, lipid peroxidation, and ferroptosis in MCAO mice. TSN modulated the infiltration of immune cells in MCAO mice in vivo and T cell differentiation in vitro. TSN directly interacted with ACSL4 and suppressed the ACSL4/LPCAT3 axis to regulate lipid metabolism and ferroptosis.

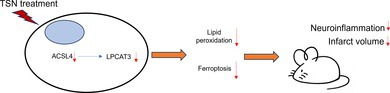

## 1 Introduction

Stroke arises from the obstruction of blood vessels due to thrombi or emboli, standing as the second foremost cause of death globally [1]. The interruption of blood flow initiates a series of pathological processes such as excitotoxicity, oxidative stress, and neuroinflammation, all synergistically leading to neuronal damage and cellular demise. Notably, stroke results in permanent impairments in 80% of survivors, with annual global estimates of 5.9 million fatalities and 102 million disability cases [2]. Cerebral ischemia constitutes 87% of all stroke cases, characterized by inadequate oxygen and nutrient delivery that leads to significant brain tissue damage [3]. Following vascular occlusion, the neuroinflammatory cascade is promptly initiated and exhibits a dual function. In the acute phase, it aggravates damage by activating microglia and recruiting peripheral immune cells that secrete inflammatory cytokines, which exacerbates blood–brain barrier disruption, cerebral edema, and apoptotic neuronal death [4]. Conversely, in the later phase, inflammation aids recovery by removing debris and encouraging tissue repair, with the timing and context critically influencing both injury severity and functional restoration. Current therapeutic strategies emphasize prompt reperfusion via thrombolytics such as tissue plasminogen activator or mechanical thrombectomy, but a narrow treatment window and the risk of hemorrhagic complications constrain their use [5]. Thus, elucidating the molecular basis for neuroinflammation and developing interventions to counter its pathological aspects may advance cerebral ischemia therapeutics.

Ferroptosis is an iron‐dependent form of regulated cell death characterized by the accumulation of lethal lipid peroxides [6]. Since its formal identification in 2012, ferroptosis has emerged as a critical process in various physiological and pathological contexts, including cancer, neurodegenerative diseases, and ischemia–reperfusion injury [7, 8, 9]. The core mechanism of ferroptosis involves the peroxidation of polyunsaturated fatty acid (PUFA)‐containing phospholipids within cellular membranes [10]. This process is driven by iron, which catalyzes the Fenton reaction and activates enzymes such as lipoxygenases. Recent studies have uncovered that ACSL4 and LPCAT3 facilitate the incorporation of PUFAs into membrane phospholipids, thereby enhancing cellular susceptibility to ferroptosis [11]. Accumulating studies indicate that ferroptosis has emerged as a critical pathological mechanism in cerebral ischemia [12]. In ischemic stroke, disrupted blood flow triggers iron accumulation, reactive oxygen species (ROS) overproduction, and glutamate excitotoxicity, collectively promoting ferroptosis. Notably, ferroptosis contributes to neuronal loss, blood–brain barrier disruption, and neuroinflammation, thus exacerbates ischemic injury. Therapeutic strategies targeting ferroptosis, including iron chelators, lipophilic antioxidants, and inhibitors of lipid peroxidation, show promise to mitigate ischemic damage in preclinical models [13]. However, clinical translation requires further exploration of tissue‐specific mechanisms and optimal intervention timing.

Toosendanin (TSN), a triterpenoid compound derived from *Melia toosendan* Sieb et al., Zucc., has been historically used in traditional Chinese medicine for its analgesic and anthelmintic properties [14]. Beyond its initial recognition as an insecticidal agent, recent studies have revealed a wide range of pharmacological activities of TSN, including anticancer, anti‐inflammatory, and neurobiological effects [15]. Its mechanism entails the modulation of ion channels, such as potassium channel inhibition and L‐type calcium channel facilitation, thereby altering cellular excitability and signal transduction [16, 17]. Additionally, TSN functions as a selective neuromuscular blocker by regulating neurotransmitter release, a characteristic that also accounts for its antagonistic effect against botulinum neurotoxin [18]. In cancer research, it promotes apoptosis and autophagy in models including glioblastoma and hepatocellular carcinoma via upregulation of ER*β* and p53 [19, 20]. Recent investigations further demonstrate its immunomodulatory capacity, exemplified by suppressing M1 macrophage polarization to contribute to its anti‐inflammatory action in experimental colitis [21]. In glioblastoma, TSN interacts with Hck and Lyn to reverse macrophage‐mediated immunosuppression and overcome glioblastoma resistance to CAR‐T immunotherapy [22]. Owing to its multitargeted bioactivity and unique mechanisms, TSN presents considerable potential for therapeutic development in cerebral ischemia.

In the present study, mouse MCAO and OGD/R models were constructed to evaluate TSN's impact on cerebral ischemia–reperfusion injury. We found that TSN administration attenuated post‐stroke brain injury, neuroinflammation, and promoted long‐term functional recovery in MCAO mice by suppressing oxidative stress, ferroptosis, and modulating immune cell infiltration. Mechanistically, TSN directly bound with ACSL4 and suppressed the ACSL4/LPCAT3 axis to regulate lipid peroxidation and ferroptosis. Our results revealed a novel role of TSN in cerebral ischemia–reperfusion injury.

## 2 Materials and Methods

### 2.1 Transient Middle Cerebral Artery Occlusion (MCAO) Model, OGD/R Procedure and Drug Treatment

Animal studies were reviewed and approved by the Institutional Animal Care and Use Committee of Hangzhou medical college (Approve number: 2024HZMC1302). The 6‐week‐old female C57BL/6 mice (*n* = 40) were purchased from Weitong Lihua Lab animal Tec. Co. Ltd. (China) and maintained at an average temperature of 22°C in a typical 12–12 h cycle of light/dark at 55% ± 10% humidity. A mouse model of cerebral reperfusion injury was established as previously described [23]. Briefly, C57BL/6 mice were anesthetized with 1% pentobarbital sodium (50 mg/kg) intraperitoneally and maintained on a thermostatic blanket. After exposing the left common carotid artery (CCA), external carotid artery (ECA), and internal carotid artery (ICA) via an incision, the CCA was ligated with a surgical nylon monofilament, while the ECA was ligated distally and the ICA‐ECA bifurcation proximally. To induce middle cerebral artery occlusion, a 6.0 mm monofilament was advanced from the ECA through the CCA bifurcation into the ICA intracranial segment, with successful occlusion confirmed by a ≥ 70% reduction in regional cerebral blood flow (rCBF, monitored via Laser Doppler flowmetry, PeriFlux System 5000, PERIMED, Sweden). The monofilament was removed after 60 min, whereas sham‐operated mice underwent identical surgery without filament insertion. After the MCAO model was built, mice were randomly divided into Sham, Vehicle (Veh), 0.5 mg/kg TSN, 1 mg/kg TSN, and 0.5 mg/kg TSN + 3 mg/kg RSL3 groups (*n* = 5 for each group). Mice were treated with 0.5 mg/kg TSN, 1 mg/kg TSN, 0.5 mg/kg TSN + 3 mg/kg RSL3, or vehicle immediately after ischemia and then continuously for 2 weeks daily post‐MCAO as specified. At the end of drug treatment, mice were anesthetized with 3% isoflurane and sacrificed using the neck dissociation method. To model cerebral ischemia–reperfusion injury in vitro, HT22 and SH‐SY5Y cells underwent oxygen–glucose deprivation/reoxygenation (OGD/R). Briefly, cells were cultured in glucose‐free DMEM (Gibco, USA) within a hypoxic chamber (5% CO_2_, 95% N_2_) at 37°C for 60 min, followed by 24‐h recovery in standard glucose‐containing medium under normoxic conditions. Toosendanin (#S9305, Selleck Chemicals, USA) and RSL3 (#S8155, Selleck Chemicals, USA) were prepared in 1% DMSO + 5% PEG300 + 5% Tween 80 + 89% deionized water (vehicle control). Mice received daily treatments of 0.5 mg/kg TSN, 1 mg/kg TSN, 3 mg/kg RSL3, or vehicle for 14 days post‐MCAO as specified.

### 2.2 Reverse Transcription–Quantitative PCR (RT‐qPCR)

Total RNA was extracted using TRIzol reagent (Takara, Japan), with quality confirmed by NanoDrop 2000 (Thermo Scientific, USA) measuring 260/280 nm absorbance ratios. First‐strand cDNA was synthesized using the High‐Capacity cDNA Reverse Transcription Kit (ThermoFisher Scientific #4387406), followed by RT‐qPCR in 20 μL reactions with PowerUp SYBR Green Master Mix (ThermoFisher Scientific #A25742) on a QuantStudio 6 Pro system (Applied Biosystems, USA). Gene expression was normalized to GAPDH and quantified by the 2−^ΔΔCq^ method, with primer sequences provided in Table S1.

### 2.3 Liquid Chromatography Coupled to Mass Spectrometry (LC–MS)

Long‐chain fatty acid LC–MS analysis in MCAO mice and HT22 and SH‐SY5Y cells was conducted by Shanghai Sensichip Infotech Co. (Shanghai, China) as previously described [24, 25]. Samples were incubated with 50% acetonitrile, 200 mM 3‐NPH, and 120 mM EDC (6% pyridine, 400 ng/mL acetic acid‐D3) prior to LC–MS analysis using an AB SCIEX 5500 mass spectrometer (Applied Biosystems, USA) coupled with an ACQUITY UPLC HPLC system (Waters, USA) and an ACQUITY UPLC BEH Amide Column (2.1 × 100 mm, 1.7 μm). Metabolite abundances were normalized to acetic acid‐D3 (internal standard) and cell extract weight.

### 2.4 Biotin Pull‐Down Assay

Pull‐down assays were conducted as previously reported [26]. Streptavidin magnetic beads (MedChemExpress LLC, #HY‐K0208) were pre‐blocked with 5% BSA overnight, washed three times, then resuspended in 100 μL PBST and stored at 4°C. For biotin‐TSN conjugation, 50 μL beads were mixed with 10 μM biotin or biotin‐TSN in PBS (total 1 mL), rotated at room temperature for 1 h, washed six times, and resuspended in 100 μL PBS. Then, biotin/biotin‐TSN beads were incubated with cell lysates or recombinant ACSL4 protein at 4°C for 4 h. Beads were washed three times, centrifuged at 2000 × g for 2 min, and eluted with 80 μL 1 × SDS‐PAGE buffer via boiling at 98°C for 5 min.

### 2.5 Statistical Analysis

Statistical analysis was conducted using GraphPad Prism 8.0 (GraphPad Software, USA), with one‐way ANOVA (Tukey's post hoc test) or Student's *t*‐test applied to assess group differences. *p < 0.05* was considered statistically significant. The full/complete methodology is described in Files S1.

## 3 Results

### 3.1 TSN Decreases Infarct Volume and Facilitates Function Recovery in MCAO Mice

**FIGURE 1:**
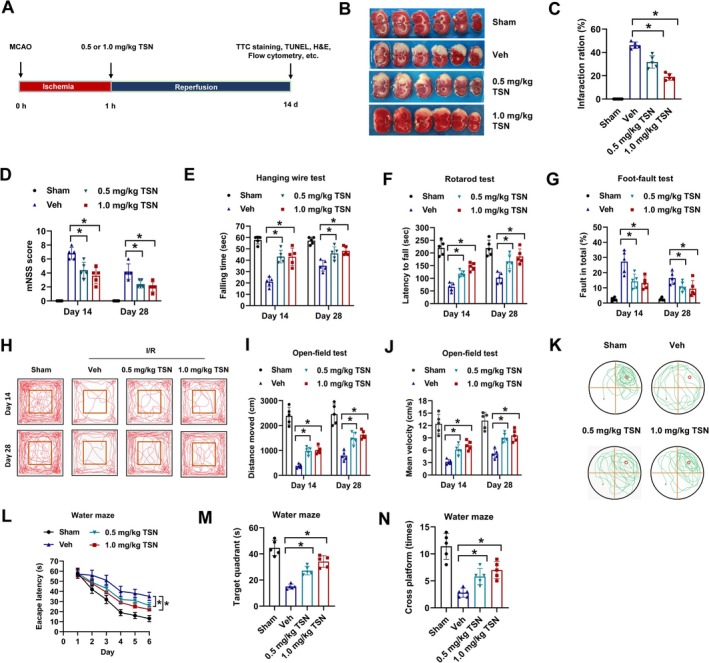
TSN decreases infarct volume and facilitates long‐term function recovery in MCAO mice. A, the experimental design of animal experiments. B, MCAO mice were treated with 0.5 mg/kg TSN, 1.0 mg/kg TSN or equal volume of Vehicle (Veh) control daily for 14 days, then infarct volume was evaluated by TT staining. Representative images (B) and infarct volumes (C) were displayed. D, neurological deficit was assessed by mNSS at day 14 and day 25 after MCAO. E‐G, Motor function recovery, including strength and coordination, was assessed through the hanging‐wire test (E), rotarod test (F), and foot‐fault test (G). H‐J, behavioral recovery in MCAO mice was assessed using the open‐field test at day 14 and day 28 post‐stroke. Motion trajectory (H), distance moved (I) and mean velocity (J) were shown. K‐N, spatial learning and memory recovery in MCAO mice were assessed with the Morris water maze at day 28 post‐stroke. Representative swimming trajectories (K), escape latency (L), time in the target quadrant (M), and platform crossing times (N) are presented. *p < 0.05.

To investigate the impact of TSN on cerebral ischemia–reperfusion injury, a transient MCAO mouse model was utilized, with post‐operative daily administration of 0.5 or 1 mg/kg TSN for 14 days (Figure 1A). TTC staining demonstrated that TSN treatment substantially diminished infarct volume compared to vehicle (Veh) controls (Figure 1B,C). Long‐term functional recovery was assessed at day 14 and day 28 post‐stroke and revealed reduced neurological deficit scores (mNSS) in TSN‐treated MCAO mice (Figure 1D). TSN treatment enhanced motor function by improving strength and coordination, as indicated by prolonged fall latencies in hanging wire and rotarod tests (Figure 1E,F), and decreased contralateral forelimb foot‐faults (Figure 1G). Behavioral analyses via open field test showed that vehicle‐treated MCAO mice had impaired exploration, reduced movement distance, and lower velocity compared to sham groups, which were partially rescued by TSN intervention (Figure 1H–1I). Further, spatial learning and memory evaluated through Morris water maze testing revealed that TSN‐treated mice had shorter escape latencies, increased platform crossings, and more time in the target quadrant, denoting cognitive recovery (Figure 1K–1N). We also tested this in early time points of day 3 and day 7 post‐stroke. Post‐stroke TSN treatment also facilitated short‐term functional recovery (Figure S1A) and motor function recovery in hanging wire (Figure S1B), rotarod tests (Figure S1D), and open field test (Figure S1G). Overall, these results indicate that post‐stroke TSN administration attenuates infarct size and facilitates functional restoration in MCAO mice.

### 3.2 TSN Attenuates Poststroke Brain Damage and Neuroinflammation in MCAO Mice

**FIGURE 2:**
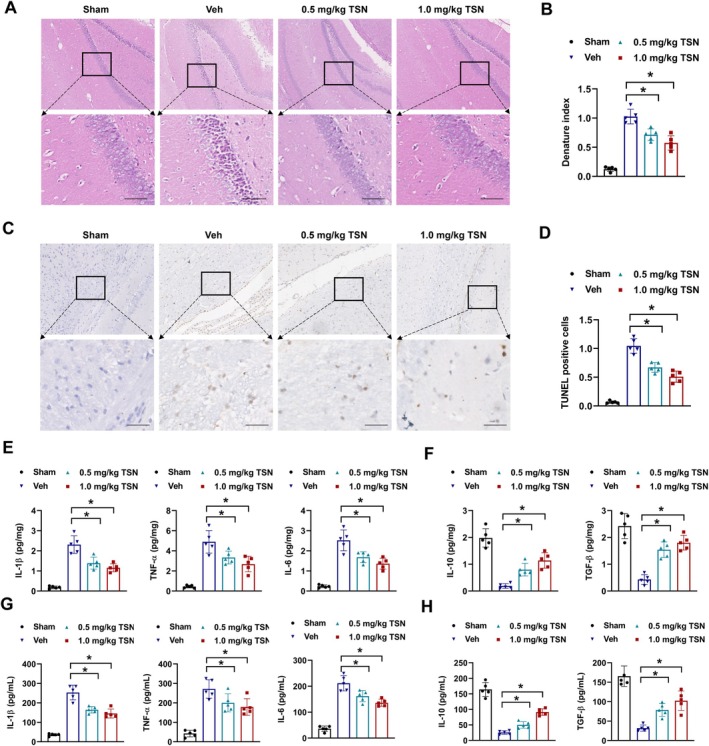
TSN attenuates poststroke brain damage and neuroinflammation in MCAO mice. A‐B, brain tissue histopathology was assessed with H&E staining. Representative images were shown in (A), and the denatured cell index was presented in (B). C‐D, cell apoptosis was assessed by TUNEL staining. Representative photos (C) and percent of TUNEL positive cells (D) were shown. (E‐H) the levels of pro‐inflammatory cytokines (IL‐1*β*, TNF‐α, and IL‐16) and anti‐inflammatory cytokines (IL‐10 and TGF‐*β*) were evaluated by ELISA assays in brain tissues (E and F) and peripheral blood (G and H). *p < 0.05.

To evaluate the therapeutic effect of TSN on cerebral ischemia–reperfusion injury, neuronal damage and neuroinflammation were assessed in MCAO mice. Immunohistochemical analysis revealed that vehicle‐treated MCAO mice exhibited significant histopathological alterations, including neuronal loss, nuclear condensation, and vacuolation compared to the sham group, whereas TSN treatment markedly ameliorated these pathological changes (Figure 2A,B). TUNEL assays further confirmed reduced neuronal apoptosis, with TSN‐treated mice showing significantly fewer TUNEL‐positive cells compared to vehicle groups (Figure 2C,D). Neuroinflammatory profiling demonstrated elevated levels of pro‐inflammatory cytokines (IL‐1*β*, TNF‐α, and IL‐16) and reduced levels of anti‐inflammatory cytokines (IL‐10 and TGF‐*β*) in brain tissues of vehicle‐treated MCAO mice, which were substantially reversed by TSN treatment (Figure 2E,F). Similar findings were also demonstrated in peripheral blood of MCAO mice (Figure 2G,H). Collectively, these findings indicate that TSN effectively mitigates post‐stroke neuronal damage and inflammatory responses in the MCAO mice model.

### 3.3 TSN Alleviates Neural Oxidative Stress and Ferroptosis in MCAO Mice or In Vitro After OGD/R

**FIGURE 3:**
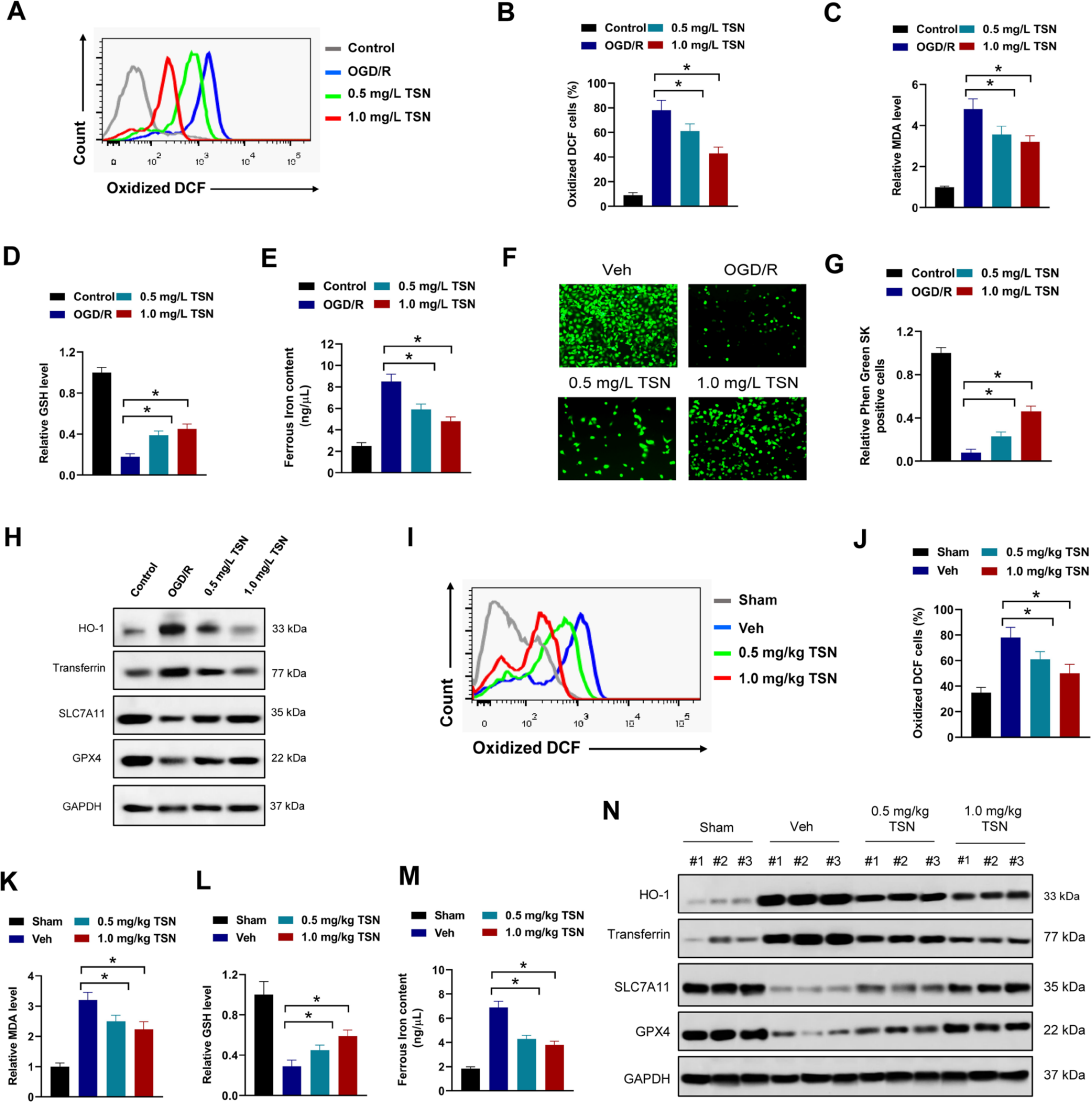
TSN alleviates oxidative stress and neural ferroptosis in MCAO mice or in vitro after OGD/R. A‐B, HT22 cells were treated with 0.5 mg/mL TSN, 1.0 mg/mL TSN or equal volume of DMSO as indicated for 24 h post OGD/R, then oxidized DCF was assessed by flow cytometry. Representative histogram (A) and percentage of oxidized DCF positive cells (B) were shown. C‐E, MDA (C), GSH (D) and ferrous iron content (E) were measured in HT22 cells treated as indicated 24 h post OGD/R. F‐G, HT22 cells were treated with 0.5 mg/mL TSN, 1.0 mg/mL TSN or equal volume of DMSO as indicated for 24 h post OGD/R, then incubated with 5 μM Phen Green SK probe to detect intracellular chelatable iron. Representative images (F) and relative Phen Green SK positive cells (G) were shown. H, HT22 cells were treated with 0.5 mg/mL TSN, 1.0 mg/mL TSN or equal volume of DMSO as indicated for 24 h post OGD/R, then collected lysates for western blot. I‐M, MCAO mice were exposed to 0.5 mg/kg TSN, 1.0 mg/kg TSN or equal volume of Vehicle (Veh) control daily for 14 days post‐stroke. Oxidized DCF was assessed by flow cytometry. Representative histogram (I) and percentage of oxidized DCF positive cells (J) were shown. MDA (K), GSH (L) and ferrous iron content (M) were measured. Ferroptosis‐related proteins were evaluated by western blot (N). *p < 0.05.

**FIGURE 4:**
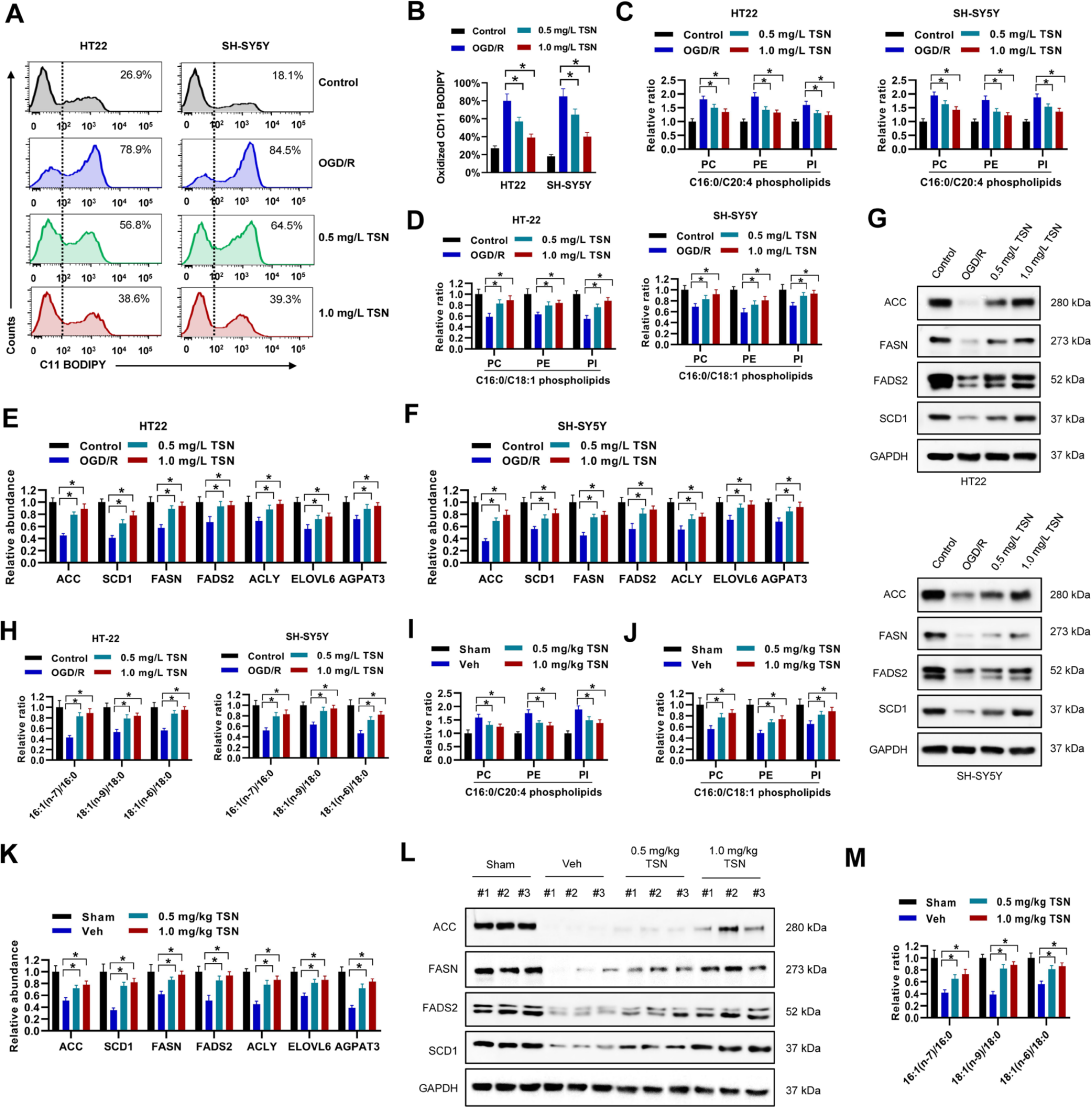
TSN promotes lipid metabolism remodeling in MCAO mice or in vitro after OGD/R. A‐B, HT22 and SH‐SY5Y cells were treated with 0.5 mg/mL TSN, 1.0 mg/mL TSN, or equal volume of DMSO as indicated for 24 h post OGD/R, then incubated with C11 BODIPY for cytometry. Representative histogram (A) and percentage of oxidized C11 BODIPY positive cells (B) were shown. C‐D, the contents of C16:0/C20:4 PL‐PUFA (C) and C16:0/C18:1 PL‐MUFA (D) in PC, PE, and PI of HT22 and SH‐SY5Y cells were assessed by LC–MS. E‐G, expression of indicated genes in HT22 and SH‐SY5Y cells were evaluated by RT‐qPCR (E‐F) and western blot (G). H, the ratio between 16:1(n‐7), 18:1(n‐9), and 18:1(n‐6) MUFAs, and 16:0 and 18:0 SFAs in HT22 and SH‐SY5Y cells were assessed by LC–MS. I‐M, MCAO mice were exposed to 0.5 mg/kg TSN, 1.0 mg/kg TSN, or equal volume of Vehicle (Veh) control daily for 14 days post‐stroke. The contents of C16:0/C20:4 PL‐PUFA (I) and C16:0/C18:1 PL‐MUFA (J) in PC, PE, and PI of brain tissues of MCAO mice were evaluated by LC–MS. The levels of indicated genes were evaluated by RT‐qPCR (K) or western blot (L). The ratio between 16:1(n‐7), 18:1(n‐9), and 18:1(n‐6) MUFAs, and 16:0 and 18:0 SFAs were assessed by LC–MS. *p < 0.05.

To elucidate the molecular mechanism by which TSN protects against cerebral ischemia–reperfusion injury, an in vitro oxygen–glucose deprivation/reoxygenation (OGD/R) model using HT22 mouse hippocampal neurons was constructed. Accumulated studies indicate that ferroptosis plays a vital role in cerebral ischemia–reperfusion injury [27]. ROS generation, lipid peroxidation, glutathione (GSH) depletion, and increasing cellular iron levels are critical events in ferroptosis. TSN treatment significantly mitigated OGD/R‐induced oxidative stress, as evidenced by a reduction in oxidized DCF‐positive cells measured by CM‐H2DCFDA fluorescence in flow cytometry (Figure 3A,B). Furthermore, TSN attenuated lipid peroxidation by decreasing malondialdehyde (MDA) levels (Figure 3A) and 4‐HNE (Figure S2A), and restored the antioxidant capacity by increasing GSH in HT22 cells after OGD/R (Figure 3D). Meanwhile, TSN treatment significantly reduced cellular ferrous iron in HT22 cells after OGD/R (Figure 3E). The fluorescent probe Phen Green SK, capable of detecting various metal ions including Fe^2+^, was employed to monitor intracellular chelatable iron by its iron‐dependent fluorescence quenching [28]. In our study, TSN increased the number of Phen Green SK‐positive cells in HT22 cells after OGD/R, suggesting a decrease in intracellular iron (Figure 3F,G). RSL3 and Erastin are well‐known inducers of cell ferroptosis. TSN treatment suppressed RSL3‐ and Erastin‐induced cell death in HT22 cells (Figure S2B). Western blot analysis revealed that OGD/R upregulated pro‐ferroptotic proteins such as HO‐1 and transferrin, and downregulated key anti‐ferroptotic regulators, including GPX4 and SLC7A11. However, these effects were substantially reversed by TSN treatment (Figure 3H). Similarly, RSL3‐ and Erastin‐induced upregulation of pro‐ferroptotic proteins (HO‐1 and transferrin) and downregulation of key anti‐ferroptotic regulators (GPX4 and SLC7A11) were also reversed by TSN treatment (Figure S2C). The above results suggested that TSN might suppress neural oxidative stress and ferroptosis in vitro after OGD/R. Consistent with the in vitro findings, the protective effects of TSN against oxidative stress and ferroptosis were further validated in the brains of MCAO mice. TSN administration significantly attenuated the stroke‐induced increase in oxidized DCF‐positive cells, indicating a reduction in overall ROS levels (Figure 3I,J). Furthermore, TSN treatment notably decreased the concentrations of MDA and cellular ferrous iron and elevated the level of GSH, collectively demonstrating its efficacy in mitigating lipid peroxidation and restoring redox balance (Figure 3K–3M). Western blot analysis confirmed that TSN effectively suppressed the expression of pro‐ferroptotic proteins HO‐1 and transferrin, and enhanced the levels of the key anti‐ferroptotic regulators GPX4 and SLC7A11 (Figure 3N). Collectively, our results indicated that TSN alleviates neural oxidative stress and ferroptosis in MCAO mice or in vitro after OGD/R.

### 3.4 TSN Modulates Infiltrating of Immune Cells in MCAO Mice and T Cell Differentiation In Vitro

Given that TSN administration alleviated neuroinflammation in MCAO mice, we hypothesized its influence on immune cell infiltration within the ischemic brain. The gating strategy of flow cytometry analysis was shown in Figure S3. Flow cytometric analysis at day 7 and day 14 post‐stroke demonstrated that TSN treatment significantly reduced the infiltration of CD45^+^ leukocytes, CD3^+^ T cells, CD3^+^CD4^+^ T cells, CD3^+^CD8^+^ T cells, CD3^−^CD19^−^ myeloid cells, Ly6G^+^CD11b^+^ neutrophils, and Ly6C^+^CD11b^+^ monocytes, while showing no marked effect on CD3^−^CD19^+^ B cells (Figure S4A). Further subset analysis revealed that TSN markedly decreased the proportions of infiltrating pro‐inflammatory IFN‐γ^+^ Th1 and IL‐17A^+^ Th17 cells and increased the proportions of anti‐inflammatory IL‐4^+^ Th2 and FoxP3^+^ Treg cells (Figure S4B–G). These results indicated that TSN treatment modulated the immune response by altering the infiltrating of immune cells in MCAO mice. To assess the impact of TSN on T cell differentiation and activation in vitro, naïve T cells isolated from mouse PBMCs were activated with anti‐CD3 and anti‐CD28 antibodies and indirectly co‐cultured via a transwell system with HT22 cells, which had been pre‐exposed to OGD/R with or without TSN treatment, under Th1, Th2, Th17, or Treg polarizing conditions. Under Th1‐polarizing conditions, co‐culture with TSN‐treated HT22 cells resulted in a reduced percentage of IFN‐γ^+^ Th1 cells compared to co‐culturing with vehicle‐treated HT22 cells (Figure S5A,B). Similarly, co‐culture with TSN‐treated HT22 cells promoted the differentiation of IL‐4^+^ Th2 cells and CD25^+^Foxp3^+^ Tregs while suppressing the differentiation of IL‐17A^+^ Th17 cells (Figure S5C–H). To evaluate the influence of TSN on the proliferation of specific CD4^+^ T helper subsets, Th1, Th2, Th17, and Treg cells were isolated from the peripheral blood of C57BL/6 mice, labeled with CFSE, and exposed to conditioned medium from HT22 cells that had undergone OGD/R with or without TSN treatment. Co‐cultured with conditioned medium from TSN‐treated HT22 cells reduced the percentage of CFSE negative Th1 and Th17 cells and increased the percentage of CFSE negative Th2 and Treg cells compared with cells co‐cultured with conditioned medium from vehicle‐treated HT22 cells, suggesting reduced proliferation of Th1 and Th17 cells and increased proliferation of Th2 and Treg cells (Figure S5I,J). Collectively, these data demonstrate that TSN modulates infiltrating of immune cells in MCAO mice and T cell differentiation in vitro.

### 3.5 TSN Promotes Lipid Metabolism Remodeling in MCAO Mice or In Vitro After OGD/R

Ferroptosis is critically driven by lipid peroxidation [29, 30]. In this study, we employed the C11 BODIPY probe to assess lipid peroxidation in HT22 cells and human neuroblastoma cell line SH‐SY5Y, which is a common model for cerebral ischemia‐injury research. Our results demonstrated that TSN treatment markedly reduced oxidized C11 BODIPY levels in both cell types following OGD/R, indicating a suppression of lipid peroxidation (Figure 4A,B). Since phospholipid polyunsaturated fatty acids (PL‐PUFAs) are known to propagate peroxidation and promote ferroptosis, whereas phospholipid monounsaturated fatty acids (PL‐MUFAs) can inhibit it by displacing PL‐PUFAs from plasma membranes [29], we analyzed specific phospholipid species. TSN treatment significantly decreased the content of C16:0/C20:4 PL‐PUFA in phosphatidylcholine (PC), phosphatidylethanolamine (PE), and phosphatidylinositol (PI) in HT22 and SH‐SY5Y cells after OGD/R (Figure 4C). Conversely, the levels of C16:0/C18:1 PL‐MUFA in these phospholipids were notably increased (Figure 4D). Oxidized PUFA‐PE such as PE‐(18:0/20:4‐OOH) and PE‐(18:0/22:4‐OOH), which are lipid peroxidation products central to ferroptosis, were also downregulated by TSN treatment (Figure S6A,B). Besides, other lipid classes such as sphingolipids (Cer(d18:1/16:0), Cer(d18:1/18:0), Cer(d18:1/24:1)) and triglycerides (TAG (16:0/18:0/20:4), TAG (16:0/16:0/22:6)) were not affected by TSN treatment (Figure S6A,B). Further investigation into key enzymes of *de novo* lipid synthesis via qRT‐PCR and western blot revealed that TSN upregulated the mRNA expression of *ACC*, *SCD1*, *FASN*, *FADS2*, *ACLY*, *ELOVL6*, and *AGPAT3* in both cell lines (Figure 4E,F), and increased the protein levels of ACC, FASN, FADS2, and SCD1 (Figure 4G). The elevated ratio of 16:1(*n*‐7), 18:1(*n*‐9), and 18:1(*n*‐6) MUFAs to 16:0 and 18:0 saturated fatty acids (SFAs) following TSN treatment particularly highlighted the role of SCD1 as a key enzyme for MUFA generation [31], confirming enhanced SCD1 activity (Figure 4H). These findings were corroborated in an in vivo MCAO mouse model, where TSN similarly reduced C16:0/C20:4 PL‐PUFA and increased C16:0/C18:1 PL‐MUFA content in brain tissue PC, PE, and PI (Figure 4I,J). Consistent with the cellular data, TSN elevated the mRNA expression of the aforementioned synthesis enzymes (Figure 4K) and the protein levels of ACC, FASN, FADS2, and SCD1 in the brains of MCAO mice (Figure 4L), along with increasing the MUFA to SFA ratio (Figure 4M). Collectively, these data indicate that TSN induces a remodeling of lipid metabolism in both in vitro OGD/R models and in vivo MCAO mice, shifting the balance from pro‐ferroptotic PUFAs toward anti‐ferroptotic MUFAs.

### 3.6 ACSL4 Is a Direct TSN Binding Target

**FIGURE 5:**
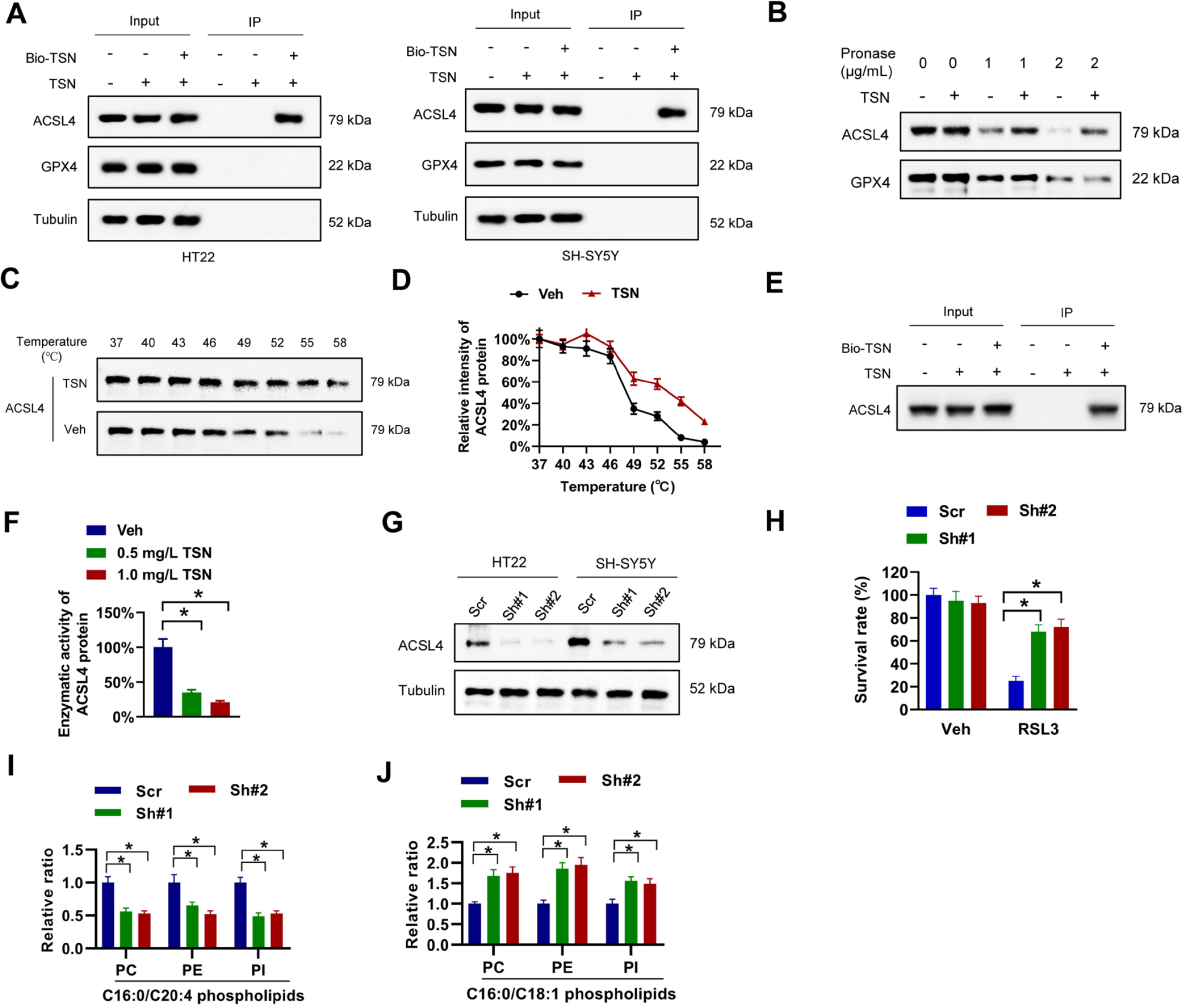
ACSL4 is a direct TSN binding target. A, the cell lysates of HT22 and SH‐SY5Y cells were incubated with 200 μM TSN or biotin‐labeled TSN (Bio‐TSN) for 2 h, then pulled down by streptavidin beads. B, HT22 cell lysates were incubated with or without 1.0 mg/mL TSN for 2 h followed by pronase treatment at varying concentrations, then ACSL4 and GPX4 expression levels were analyzed via western blot. C‐D, HT22 cell lysates were incubated with 1.0 mg/mL TSN or equal volume of DMSO (Veh), then treated with increasing melting temperature. The levels of ACSL4 were determined by western blot. E, recombinant ACSL4 protein was incubated with 200 μM TSN or biotin‐labeled TSN (Bio‐TSN) for 2 h, then pulled down by streptavidin beads. F, recombinant ACSL4 protein was incubated with 0.5 mg/mL TSN, 1.0 mg/mL TSN or equal volume of DMSO for 10 min at 37°C, then the enzymatic activity of ACSL4 was assessed via AA‐CoA generation quantification from AA. G, HT22 and SH‐SY5Y cells were introduced with shRNA targeting ACSL4, then collected lysates for western blot. H, HT22 cells introducing with ACSL4 Sh#1, Sh#2 or a scramble shRNA (Scr) were treated with 5 μM RSL3 or equal volume of DMSO for 72 h, then survival cells were evaluated by cell viability assay. I‐J, the contents of C16:0/C20:4 PL‐PUFA (I) and C16:0/C18:1 PL‐MUFA (J) in PC, PE, and PI of HT22 cells introducing with ACSL4 Sh#1, Sh#2 or a scramble shRNA (Scr) were evaluated by LC–MS. *p < 0.05.

To elucidate the specific mechanism by which TSN inhibits ferroptosis, we sought to identify its potential biological target. A biotin‐tagged TSN (Bio‐TSN) was designed and synthesized as previously described [32]. Using this synthetically designed Bio‐TSN probe for affinity pulldown assays with HT22 cell lysates and LC–MS/MS analysis, ACSL4 emerged as the highest‐intensity protein candidate. Subsequent validation via biotin‐streptavidin pulldown confirmed that Bio‐TSN specifically interacted with ACSL4 but not GPX4, in both HT22 and SH‐SY5Y cells (Figure 5A). This interaction was further substantiated using drug affinity‐responsive target stability (DARTS) and cellular thermal shift assays (CETSA), which demonstrated that TSN binding stabilized ACSL4 against proteolytic degradation and enhanced its thermostability (Figure 5B–5D). Direct binding was confirmed by incubating Bio‐TSN with recombinant ACSL4 protein (Figure 5E), and functional assessment revealed that TSN markedly suppressed ACSL4 enzymatic activity (Figure 5F). ACSL4 is a key enzyme in lipid metabolism and mediates ferroptosis through its role in esterifying PUFAs to acyl‐CoA. In our study, we knocked down ACSL4 using shRNAs in HT22 and SH‐SY5Y cells (Figure 5G), which enhanced cell survival under ferroptosis induced by RSL3 (Figure 5H) and shifted phospholipid profiles by reducing pro‐ferroptotic C16:0/C20:4 PL‐PUFA and increasing anti‐ferroptotic C16:0/C18:1 PL‐MUFA content (Figure 5I,J). These findings collectively establish ACSL4 as a direct functional target of TSN.

### 3.7 TSN Suppresses ACSL4/LPCAT3 Axis to Regulate Lipid Metabolism and Ferroptosis

**FIGURE 6:**
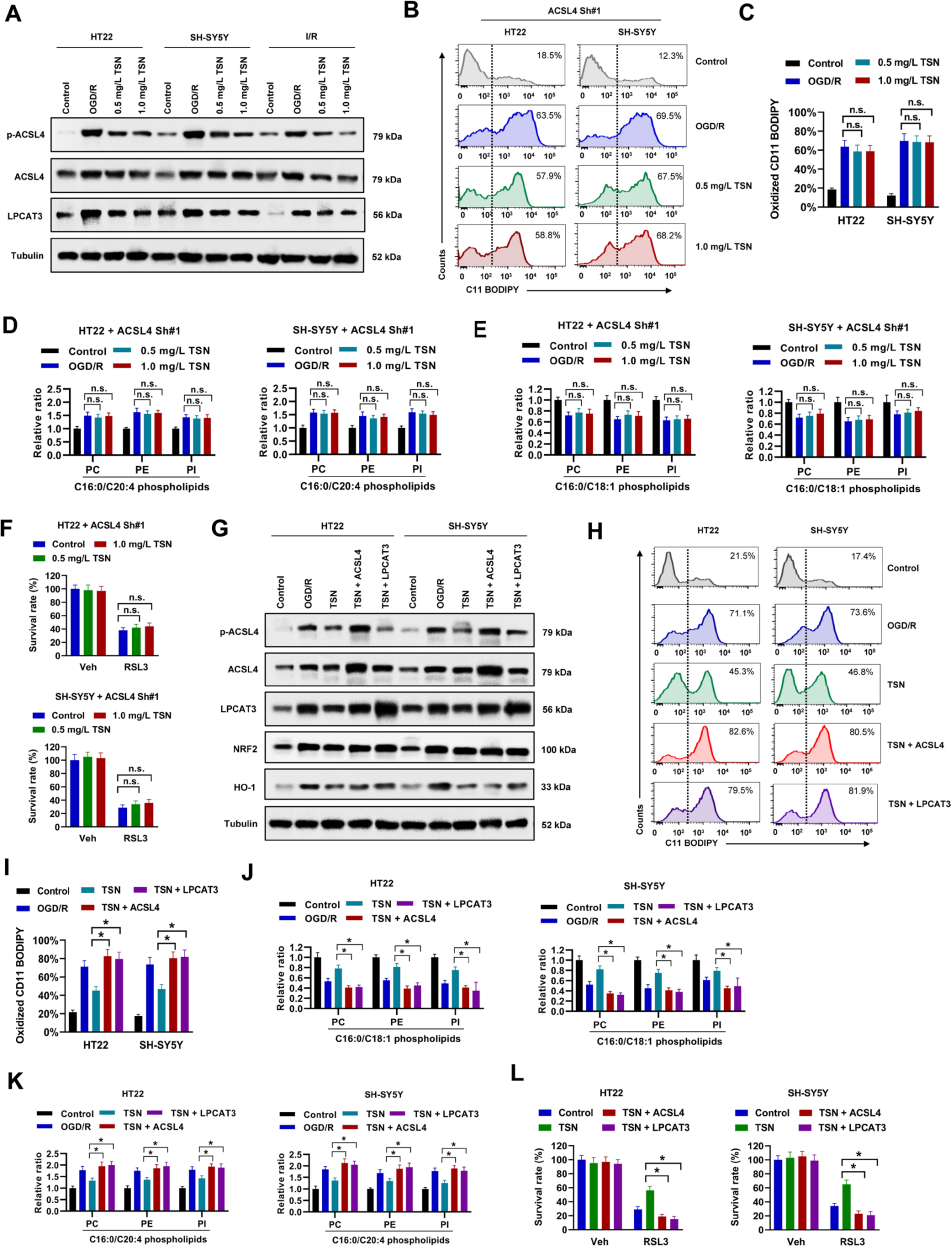
TSN suppresses ACSL4/LPCAT3 axis to regulate lipid metabolism and ferroptosis. A, cell lysates from TSN‐treated HT22 and SH‐SY5Y cells or MCAO mice were used for western blot. B‐E, HT22 and SH‐SY5Y cells introducing ACSL4 Sh#1 were treated with 0.5 mg/mL TSN, 1.0 mg/mL TSN or equal volume of DMSO as indicated for 24 h post OGD/R. Cells were incubated with C11 BODIPY for cytometry. Representative histogram (B) and percentage of oxidized C11 BODIPY positive cells (C) were shown. The contents of C16:0/C20:4 PL‐PUFA (D) and C16:0/C18:1 PL‐MUFA (E) in PC, PE, and PI were evaluated by LC–MS. F, HT22 and SH‐SY5Y cells introducing ACSL4 Sh#1 were treated with 0.5 mg/mL TSN, 1.0 mg/mL TSN, 5 μM RSL3 or equal volume of DMSO as indicated for 72 h, then survival cells were evaluated by cell viability assay. G‐K, HT22 and SH‐SY5Y cells were treated with 1.0 mg/mL TSN with/without ACSL4 or LPCAT3 overexpression as indicated for 24 h post OGD/R. Expression of indicated genes was evaluated by western blot (G). Cells were incubated with C11 BODIPY for cytometry. Representative histogram (H) and percentage of oxidized C11 BODIPY positive cells (I) were shown. The contents of C16:0/C20:4 PL‐PUFA (J) and C16:0/C18:1 PL‐MUFA (K) in PC, PE, and PI were evaluated by LC–MS. L, HT22 and SH‐SY5Y cells introducing/without ACSL4 or LPCAT3 expression lentivirus were treated with 1.0 mg/mL TSN, 5 μM RSL3 or equal volume of DMSO as indicated for 72 h, then survival cells were evaluated by cell viability assay. *p < 0.05.

Recent evidence demonstrates that Thr328 phosphorylation is essential for ACSL4 enzymatic activation [33], while LPCAT3 acts as a downstream enzyme cooperating with ACSL4 in lipid remodeling [34]. Our findings revealed that TSN treatment reduced ACSL4 phosphorylation and LPCAT3 protein levels in both HT22 and SH‐SY5Y cells, as well as brain tissues of MCAO mice (Figure 6A). To investigate whether TSN regulates lipid remodeling and ferroptosis via ACSL4, we performed ACSL4 knockdown in these cell lines. Following ACSL4 knockdown, TSN treatment no longer affected oxidized C11 BODIPY levels, the contents of C16:0/C20:4 PL‐PUFA and C16:0/C18:1 PL‐MUFA in PC, PE, and PI, or RSL3‐induced cell death (Figure 6B–F). Conversely, overexpression of ACSL4 or LPCAT3 (Figures 6G and S7A,B) abolished the suppressive effect of TSN on oxidized C11 BODIPY levels (Figure 6H,I). Nrf2/HO‐1 pathway are involved in ferroptosis of cerebral ischemia [35, 36]. However, we found that overexpression of ACSL4 or LPCAT3 showed no influence on NRF2 expression in both HT22 and SH‐SY5Y cells (Figure 6G). Furthermore, the TSN‐induced increase in C16:0/C18:1 PL‐MUFA and decrease in C16:0/C20:4 PL‐PUFA content under OGD/R conditions were reversed by ACSL4 and LPCAT3 overexpression (Figure 6J,K). The protective effect of TSN against RSL3‐induced cell death was also abrogated by ACSL4 and LPCAT3 overexpression (Figure 6L). Collectively, these results indicate that TSN regulates lipid metabolism and ferroptosis by suppressing the ACSL4/LPCAT3 axis.

### 3.8 Inducing of Ferroptosis Abrogates the Influence of TSN on Immune Infiltration and Lipid Metabolism in MCAO Mice

To assess the role of ferroptosis in TSN‐mediated regulation of immune infiltration and lipid metabolism within the MCAO model, the ferroptosis inducer RSL3 was employed. TTC staining revealed that TSN administration decreased cerebral infarct volume in MCAO mice compared to vehicle controls, but this was eliminated by RSL3 co‐administration (Figure S8A,B). Furthermore, TSN significantly attenuated the infiltration of various immune cells, including CD45^+^ leukocytes, CD3^+^ T cells (CD4^+^ and CD8^+^ subsets), CD3^−^CD19^−^ myeloid cells, Ly6G^+^CD11b^+^ neutrophils, and Ly6C^+^CD11b^+^ monocytes; however, these suppressive effects were also abolished by RSL3 (Figure S8C). Concurrently, TSN modulated lipid composition by decreasing C16:0/C20:4 PL‐PUFA and increasing C16:0/C18:1 PL‐MUFA content in brain tissue phospholipids (PC, PE, PI), but the changes were similarly reversed upon RSL3 co‐treatment (Figure S8D,E). The TSN‐induced upregulation of key lipid metabolism genes (ACC, SCD1, FASN, FADS2, ACLY, ELOVL6, AGPAT3) was also blocked by RSL3 (Figure S8F). Collectively, these findings demonstrate that pharmacological induction of ferroptosis via RSL3 counteracts the protective effects of TSN on immune cell infiltration and lipid metabolic reprogramming (LMR) in MCAO mice.

## 4 Discussion

Since the elucidation of the TSN chemical structure in the 1980s, extensive research has explored its diverse biological activities. Emerging evidence indicates that TSN selectively inhibits acetylcholine release at nerve terminals, demonstrating its anti‐botulismic properties [14, 37]. Furthermore, it induces neuronal differentiation and process outgrowth in PC12 cells at low doses [38]. In models of acute lung injury, TSN directly binds to and inhibits mTOR activation, consequently reducing ROS production, lipid peroxidation, and endoplasmic reticulum stress both in vitro and in vivo [39]. TSN also alleviates mycoplasma pneumonia‐induced lung injury in mice by suppressing the NF‐κB‐mediated inflammatory response [40]. Additionally, TSN exhibits broad anti‐cancer efficacy across various human cancer cell types [41]. For example, TSN reverses macrophage‐mediated immunosuppression and overcomes glioblastoma resistance to CAR‐T immunotherapy [22]. Despite this wide spectrum of bioactivities, its potential impact on cerebral ischemia–reperfusion injury remains underexplored. A 2018 study by Gaowa et al., suggested that the traditional Mongolian medicine Eerdun Wurile, which contains TSN, may enhance stroke recovery by modulating gene expression in rat brains [42]. Our study now demonstrates that TSN attenuates post‐stroke brain damage and neuroinflammation and promotes long‐term functional recovery in MCAO mice, thereby revealing a novel therapeutic role for TSN in cerebral ischemia–reperfusion injury.

The complex relationship between ferroptosis and cerebral ischemia constitutes a key pathological mechanism in neuronal injury and functional deficits following ischemic stroke [43]. For instance, compounds like Astragaloside IV alleviate cerebral ischemia–reperfusion injury by suppressing ferroptosis through the P62/Keap1/Nrf2 pathway in both MCAO mice and OGD/R models [44], while Gastrodia elata‐derived neutral polysaccharide mitigates injury by inhibiting ferroptosis‐mediated neuroinflammation via NRF2/HO‐1 signaling [45]. Our study reveals that TSN exerted neuroprotective effects in cerebral ischemia/reperfusion injury by specifically targeting ferroptosis through LMR and directly binding to the ferroptosis regulator ACSL4 to shift phospholipid composition from pro‐ferroptotic PUFAs to anti‐ferroptotic MUFAs. Interestingly, TSN demonstrates context‐dependent regulation of ferroptosis, as evidenced by promoting cell death in hepatocellular carcinoma through WWOX‐mediated p53/SAT1 and NRF2/FPN1 activation [19] and inducing ferroptosis in gastrointestinal stromal tumors via NCOA4 ferritinophagy pathway regulation [46]. This functional duality highlights TSN's multifaceted therapeutic potential, which might be attributable to distinct metabolic states, redox environments, and signaling pathway activities between ischemic neurons and cancer cells.

In addition to its direct anti‐ferroptotic effects, our study revealed a complex interplay between TSN‐mediated ferroptosis inhibition and the modulation of post‐stroke neuroinflammation, where cerebral ischemia triggered a robust inflammatory response characterized by peripheral immune cell infiltration into the brain parenchyma. TSN treatment significantly reduced this infiltration and promoted a shift in CD4^+^ T helper cell polarization from pro‐inflammatory Th1 and Th17 phenotypes toward anti‐inflammatory Th2 and Treg subsets, both in vivo and in vitro. The critical link between ferroptosis inhibition and immunomodulation was established when co‐administration of the ferroptosis inducer RSL3 completely abrogated TSN's effects on immune cell infiltration and lipid metabolism, indicating that ferroptosis suppression preceded anti‐inflammatory actions, likely by preventing damage‐associated molecular pattern release from ferroptotic cells that would otherwise sustain inflammation. This crosstalk positioned TSN as a multifaceted agent via disrupting the vicious cycle between ferroptosis and neuroinflammation, which was supported by accumulated evidence highlighting ferroptosis' role in neuroinflammation regulation [43]. For instance, the Naotaifang formula attenuated OGD/R‐induced inflammation and ferroptosis via microglial M1/M2 polarization through BMP6/SMADs signaling [47], NRF2 activation ameliorating blood–brain barrier injury by modulating ferroptosis and inflammation [48], and Srs11‐92, a ferrostatin‐1 analog, improving oxidative stress and neuroinflammation through Nrf2 signaling post cerebral ischemia/reperfusion injury [49].

ACSL4 is a key enzyme in lipid metabolism, which catalyzes the activation of long‐chain fatty acids by conjugating them to coenzyme A [34]. Targeting ACSL4 may affect lipid metabolism and ferroptosis. For instance, Tongqiao Huoxue Decoction, a traditional Chinese herbal formulation, mitigates cerebral ischemia–reperfusion injury by suppressing ferroptosis via promoting ACSL4 ubiquitination and degradation [50]. Thrombin, a serine protease, initiates ferroptotic signaling by inhibiting ACSL4 to promote arachidonic acid mobilization and esterification [51]. In our study, ACSL4 was identified as a direct binding target for TSN, and our findings demonstrated that TSN binding stabilized ACSL4 and inhibited its enzymatic activity, thereby inducing a profound reprogramming of lipid metabolism. Furthermore, our results indicated that TSN suppressed the ACSL4/LPCAT3 axis to regulate lipid metabolism and ferroptosis, with the protective effects of TSN being reversed by overexpression of either ACSL4 or its collaborator LPCAT3. Indeed, the ACSL4/LPCAT3 axis plays a vital role in lipid metabolism and ferroptosis. For example, rosmarinic acid liposomes protect against cerebral ischemia injury through inhibition of TfR1 to attenuate ACSL4/LPCAT3/LOX‐mediated lipid peroxidation and ferroptosis [52], while gastrodin reduces iron accumulation and lipid peroxidation in MCAO/R rats and OGD/R‐injured PC12 cells by enhancing the xCT/GPX4 axis and repressing the ACSL4/LPCAT3 pathway [53].

## 5 Conclusion

In summary, our study demonstrated that TSN ameliorated poststroke brain injury and neuroinflammation, promoting long‐term functional recovery in MCAO mice. Mechanistically, TSN reduced neural oxidative stress and suppressed ferroptosis both in vivo and in OGD/R‐induced in vitro models. Furthermore, TSN modulated immune cell infiltration in MCAO mice and influenced T cell differentiation in vitro. ACSL4 was identified as a direct binding target of TSN, and TSN regulated lipid metabolism and ferroptosis by inhibiting the ACSL4/LPCAT3 axis. Importantly, RSL3‐induced ferroptosis abolished the protective effects of TSN on immune infiltration and lipid metabolism in MCAO mice. These findings reveal a novel role for TSN in mitigating cerebral ischemia–reperfusion injury.

## Author Contributions

All authors guaranteed the integrity of the entire study. Xinyun Li and Zhiyong Zhao conducted most of the experiments. Data were analyzed by Jingting Zhao and Xiangming Ye. Manuscript was prepared by Zhenfei Xiong and Jiejin Zhao. All authors had read and approved the manuscript.

## Funding

This work was supported by The “Pioneer and Leading Goose + X” Technology Program (2025C02200); The Zhejiang Traditional Chinese Medicine Science and Technology Program (2024ZL368); The Zhejiang Provincial Rehabilitation Medicine Association Scientific Research Special Fund Project (ZKKY2024009); The Basic scientific research project of Hangzhou Medical College (KYQN2024002); The Zhejiang Province Medical and Health Science and Technology Project (2024XY017).

## Ethics Statement

The animal studies were approved and followed with the ethical standards of the ethics committee in Hangzhou medical college (20240108101927187168).

## Conflicts of Interest

The authors declare no conflicts of interest.

## Supporting information


**Figure S1:** TSN decreases infarct volume and facilitates short‐term function recovery in MCAO mice. A, neurological deficit was assessed by mNSS at day 3 and day 7 after MCAO. B‐C, Motor function recovery, including strength and coordination, was assessed through hanging‐wire test (B), rotarod test (C), and foot‐fault test (D) at day 3 and day 7 after MCAO. H‐J, behavioral recovery in MCAO mice was assessed using the open‐field test at day 3 and day 7 post‐stroke. Motion trajectory (E), distance moved (F) and mean velocity (G) were shown. *p < 0.05.
**Figure S2:** TSN alleviates neural ferroptosis in vitro. A, HT22 cells were treated with 0.5 mg/mL TSN, 1.0 mg/mL TSN or equal volume of DMSO as indicated for 24 h post OGD/R, then 4‐HNE level was evaluated by ELISA assay. B‐C, HT22 cells were treated with 0.5 mg/mL TSN, 1.0 mg/mL TSN, 5 μM RSL3, 5 μM Erastin or equal volume of DMSO as indicated for 72 h, the survival cells was evaluated by cell viability assay (B) or collected lysates for western blot (C). *p < 0.05.
**Figure S3:** The gating strategy of flow cytometry analysis.
**Figure S4:** TSN reduces infiltrating of immune cells and immune activation in MCAO mice. A, MCAO mice were exposed to 0.5 mg/kg TSN, 1.0 mg/kg TSN or equal volume of Vehicle (Veh) control daily for 14 days post‐stroke, then infiltrating immune cells in brain tissues were evaluated by flow cytometry. B‐G, the infiltrating of IFN‐γ+ Th1, IL‐4+ Th2 (B‐C), IL‐17A+ Th17 cells (D‐E) and CD25+FoxP3+ Treg cells (F‐G) were evaluated by flow cytometry. *p < 0.05.
**Figure S5:** TSN affects T cell differentiation and activation in vitro. A‐H, naïve T cells isolated from mouse PBMCs were co‐cultured via a transwell system with HT22 cells that had been pre‐exposed to OGD/R with or without TSN treatment, then exposed to Th1, Th2, Th17 or Treg polarizing conditions. Representative plots and percentage of Th1 (A‐B), Th2 (C‐D), Th17 (E‐F) and Treg (G‐H) cells were shown. I‐J, Th1, Th2, Th17, and Treg cells were isolated from the peripheral blood of C57BL/6 mice, labeled with CFST, then exposed to conditioned medium from HT22 cells that had undergone OGD/R with or without TSN treatment. Then CFSE negative cells were evaluated by flow cytometry. Representative histogram (I) and percentage of CFSE negative cells (J) were shown. *p < 0.05.
**Figure S6:** TSN promotes lipid metabolism remodeling in vitro after OGD/R. A‐B, the contents of indicated lipid species were evaluated by LC–MS. *p < 0.05, n.s. = not significant.
**Figure S7:** Ectopic overexpression of ACSL4 and LPCAT3 in HT22 and SH‐SY5Y cells. HT22 and SH‐SY5Y cells were introduced with ACSL4 (A) or LPCAT3 (B) expression vector, then collected lysates for western blot. Empty PCDH vector was used as a negative control.
**Figure S8:** Inducing of ferroptosis abrogated the influence of TSN on immune infiltration and lipid metabolism in MCAO mice. A‐B, MCAO mice were treated with 0.5 mg/kg TSN, 3 mg/kg RSL3 or equal volume of Vehicle (Veh) control daily for 14 days, then infarct volume was evaluated by TT staining. Representative images (A) and infarct volumes (B) were displayed. C, infiltrating immune cells in brain tissues were evaluated by flow cytometry. D‐F, the contents of C16:0/C20:4 PL‐PUFA (D) and C16:0/C18:1 PL‐MUFA (E) in PC, PE, and PI of brain tissues of MCAO mice were evaluated by LC–MS. The levels of indicated genes were evaluated by RT‐qPCR (F). *p < 0.05.
**Table S1:** Sequences of primers in RT‐qPCR.

## Data Availability

The data that support the findings of this study are available on request from the corresponding author. The data are not publicly available due to privacy or ethical restrictions.
