# Supplementary material for: Toosendanin Alleviates Cerebral Ischemia/Reperfusion Injury via Inhibiting Neural Ferroptosis Through Lipid Metabolic Reprogramming in MCAO Mice
Source: CNS Neurosci Ther. 2026 May 29;32(6):e70952. doi: 10.1002/cns.70952 (PMC13240430; doi:10.1002/cns.70952)
Supplement: Supplementary file 1 — Figure S1: TSN decreases infarct volume and facilitates short‐term function recovery in MCAO mice. A, neurological deficit was assessed by mNSS at day 3 and day 7 after MCAO. B‐C, Motor function recovery, including strength and coordination, was assessed through hanging‐wire test (B), rotarod test (C), and foot‐fault test (D) at day 3 and day 7 after MCAO. H‐J, behavioral recovery in MCAO mice was assessed using the open‐field test at day 3 and day 7 post‐stroke. Motion trajectory (E), distance moved (F) and mean velocity (G) were shown. *p < 0.05. Figure S2: TSN alleviates neural ferroptosis in vitro. A, HT22 cells were treated with 0.5 mg/mL TSN, 1.0 mg/mL TSN or equal volume of DMSO as indicated for 24 h post OGD/R, then 4‐HNE level was evaluated by ELISA assay. B‐C, HT22 cells were treated with 0.5 mg/mL TSN, 1.0 mg/mL TSN, 5 μM RSL3, 5 μM Erastin or equal volume of DMSO as indicated for 72 h, the survival cells was evaluated by cell viability assay (B) or collected lysates for western blot (C). *p < 0.05. Figure S3: The gating strategy of flow cytometry analysis. Figure S4: TSN reduces infiltrating of immune cells and immune activation in MCAO mice. A, MCAO mice were exposed to 0.5 mg/kg TSN, 1.0 mg/kg TSN or equal volume of Vehicle (Veh) control daily for 14 days post‐stroke, then infiltrating immune cells in brain tissues were evaluated by flow cytometry. B‐G, the infiltrating of IFN‐γ+ Th1, IL‐4+ Th2 (B‐C), IL‐17A+ Th17 cells (D‐E) and CD25+FoxP3+ Treg cells (F‐G) were evaluated by flow cytometry. *p < 0.05. Figure S5: TSN affects T cell differentiation and activation in vitro. A‐H, naïve T cells isolated from mouse PBMCs were co‐cultured via a transwell system with HT22 cells that had been pre‐exposed to OGD/R with or without TSN treatment, then exposed to Th1, Th2, Th17 or Treg polarizing conditions. Representative plots and percentage of Th1 (A‐B), Th2 (C‐D), Th17 (E‐F) and Treg (G‐H) cells were shown. I‐J, Th1, Th2, Th17, and Treg cells were isolated fro [file CNS-32-e70952-s001.docx]

**Supplementary Files**

**Toosendanin alleviates cerebral ischemia/reperfusion injury via inhibiting neural ferroptosis through lipid metabolic reprogramming in MCAO mice**

Xinyun Li ^1^, Zhiyong Zhao ^2^, Jingting Zhao ^3^, Xiangming Ye ^3^, Zhenfei Xiong ^4#^, Jiejin Zhao ^5#^.

1. School of Rehabilitation, Hangzhou medical college, Hangzhou 311399, China.

2. Children's Hospital, Zhejiang University School of Medicine, National Clinical Research Center for Child Health, Hangzhou 310003, China.

3. Department of Rehabilitation Medicine, Zhejiang Provincial People's Hospital, Affiliated People's Hospital, Hangzhou Medical College, Hangzhou 310014, China.

4. Department Of Foot and Ankle, Department of Foot and Ankle Surgery, Xiaoshan District Hospital of Traditional Chinese Medicine, Hangzhou 310014, China.

5. Department of Rehabilitation Medicine, The First People's Hospital of Xiaoshan District, Hangzhou 312000, China.

**^#^Corresponding authors:** Jiejin Zhao and Zhenfei Xiong.

**Address:**

Jiejin Zhao. Department of Rehabilitation Medicine, No.199 Shixinnan Road, Xiaoshan District, Hangzhou 312000, Zhejiang Province, China. E-mail: zhaojiejin2018@163.com.

Zhenfei Xiong. Department of Rehabilitation Medicine, No.199 Shixinnan Road, Xiaoshan District, Hangzhou 312000, Zhejiang Province, China. E-mail: vrjp29@sina.com.

**Running title:** TSN alleviates cerebral ischemia/reperfusion injury.

**This file includes:**

Supplementary Figure 1-8;

Supplementary Table 1;

Supplementary Methods.

**Supplementary Figures**


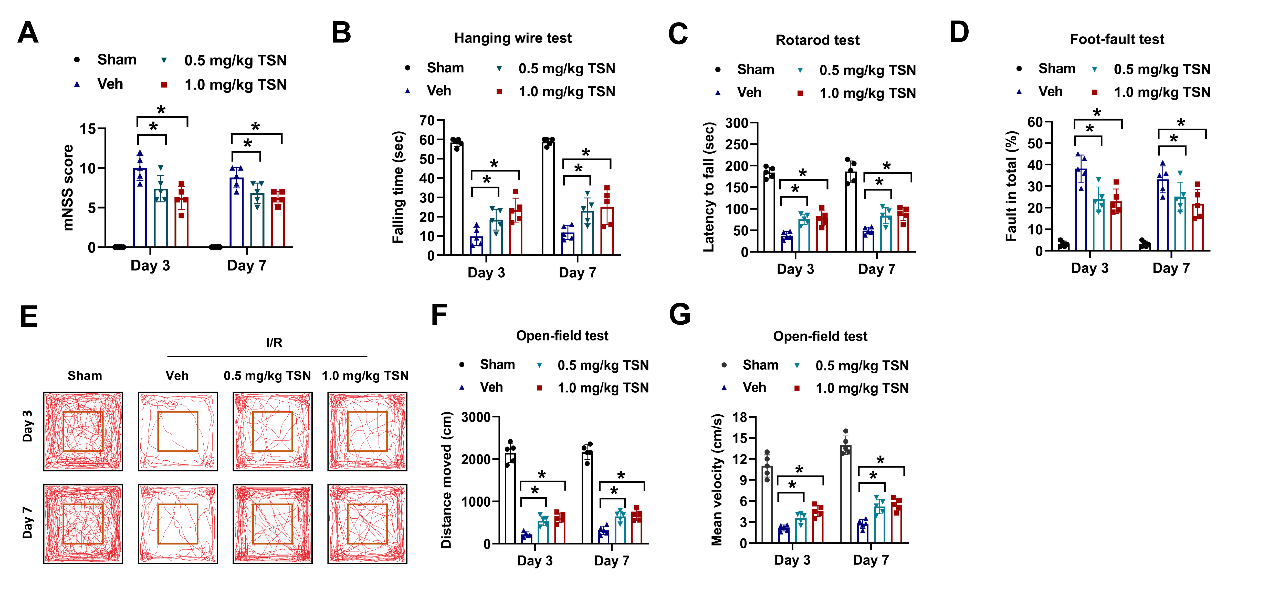


Figure S1. TSN decreases infarct volume and facilitates short-term function recovery in MCAO mice. A, neurological deficit was assessed by mNSS at day 3 and day 7 after MCAO. B-C, Motor function recovery, including strength and coordination, was assessed through hanging-wire test (B), rotarod test (C), and foot-fault test (D) at day 3 and day 7 after MCAO. H-J, behavioral recovery in MCAO mice was assessed using the open-field test at day 3 and day 7 post-stroke. Motion trajectory (E), distance moved (F) and mean velocity (G) were shown. **P*<0.05.


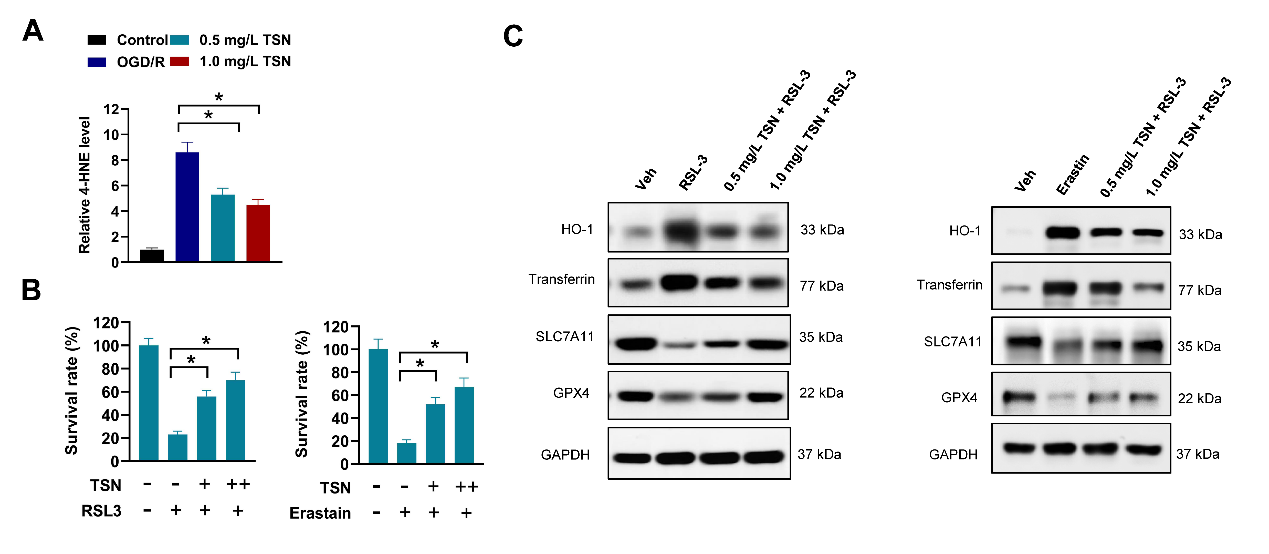


Figure S2. TSN alleviates neural ferroptosis *in vitro.* A, HT22 cells were treated with 0.5 mg/mL TSN, 1.0 mg/mL TSN or equal volume of DMSO as indicated for 24 h post OGD/R, then 4-HNE level was evaluated by ELISA assay. B-C, HT22 cells were treated with 0.5 mg/mL TSN, 1.0 mg/mL TSN, 5 μM RSL3, 5 μM Erastin or equal volume of DMSO as indicated for 72 h, the survival cells was evaluated by cell viability assay (B) or collected lysates for western blot (C). **P*<0.05.


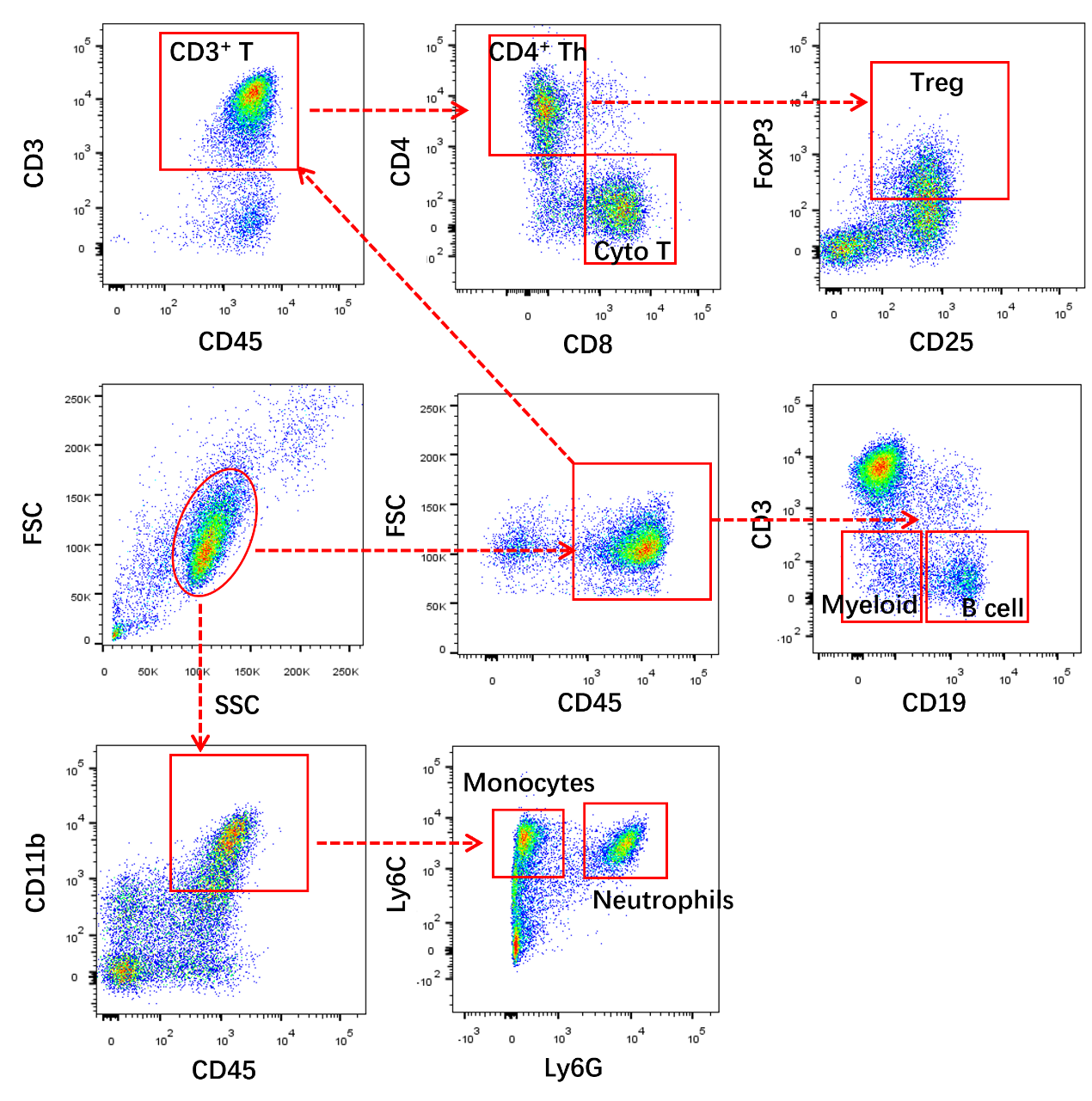


Figure S3. The gating strategy of flow cytometry analysis.


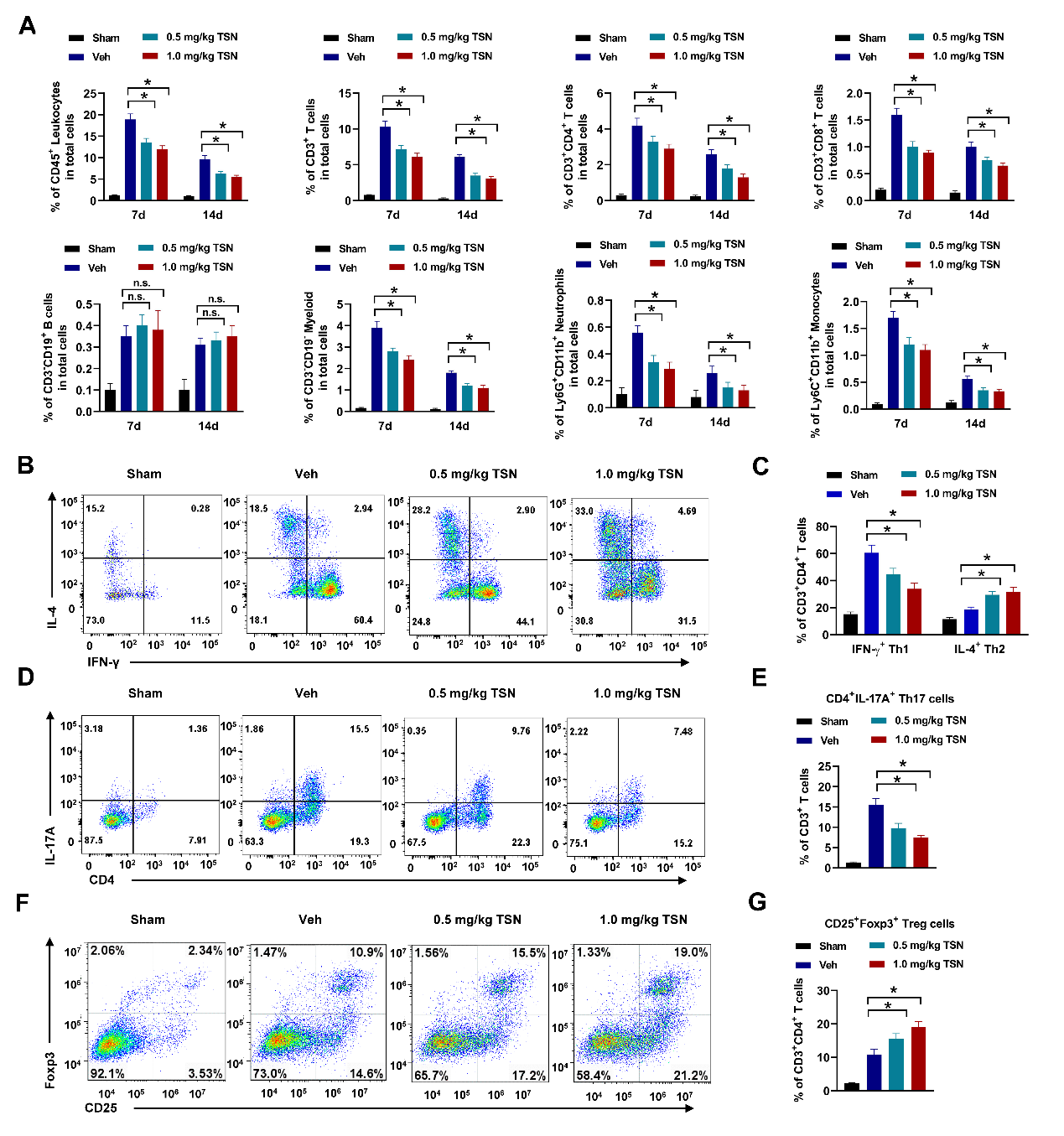


Figure S4. TSN reduces infiltrating of immune cells and immune activation in MCAO mice. A, MCAO mice were exposed to 0.5 mg/kg TSN, 1.0 mg/kg TSN or equal volume of Vehicle (Veh) control daily for 14 days post-stroke, then infiltrating immune cells in brain tissues were evaluated by flow cytometry. B-G, the infiltrating of IFN-γ^+^ Th1, IL-4^+^ Th2 (B-C), IL-17A^+^ Th17 cells (D-E) and CD25^+^FoxP3^+^ Treg cells (F-G) were evaluated by flow cytometry. **P*<0.05.


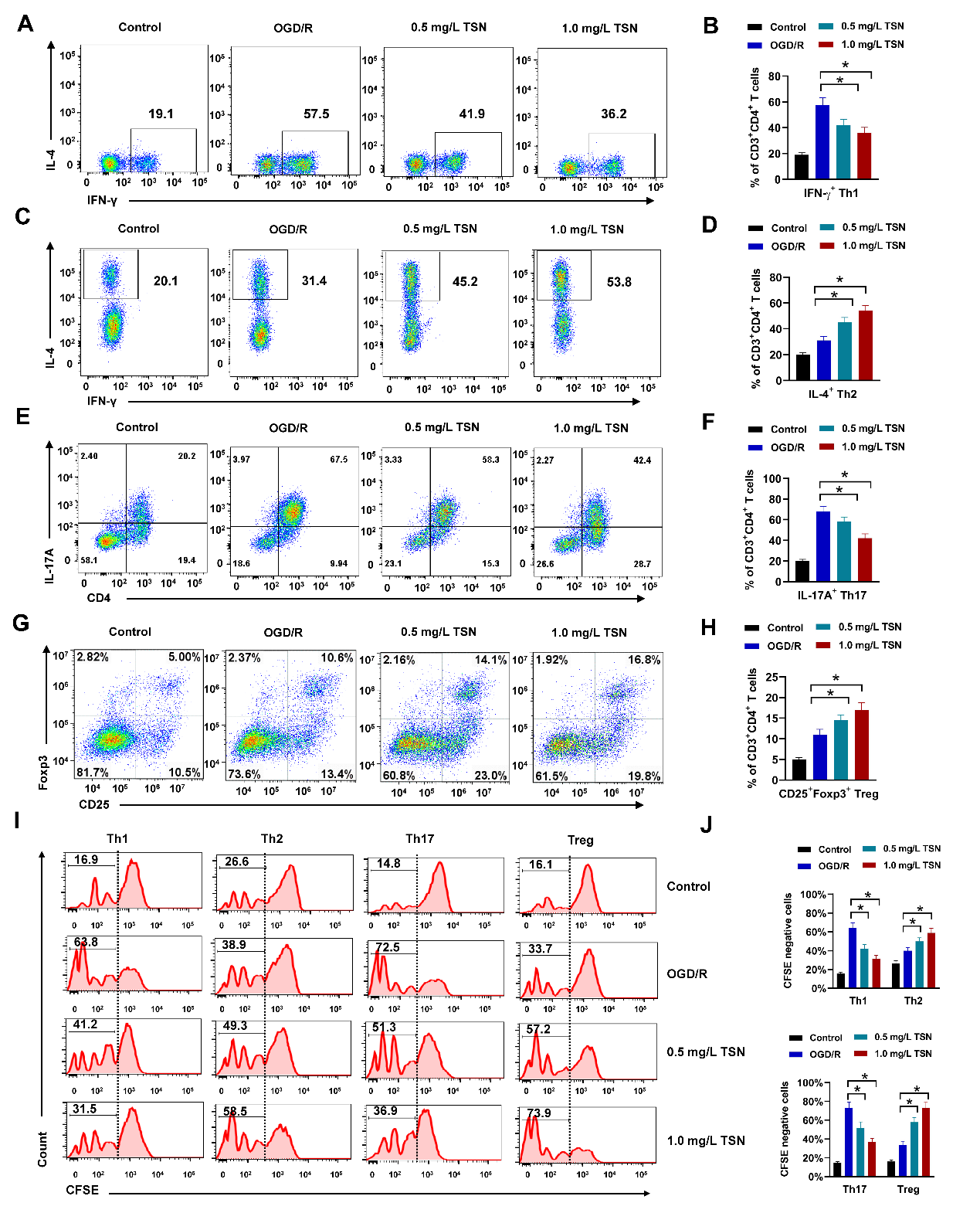


Figure S5. TSN affects T cell differentiation and activation *in vitro*. A-H, naïve T cells isolated from mouse PBMCs were co-cultured via a transwell system with HT22 cells that had been pre-exposed to OGD/R with or without TSN treatment, then exposed to Th1, Th2, Th17 or Treg polarizing conditions. Representative plots and percentage of Th1 (A-B), Th2 (C-D), Th17 (E-F) and Treg (G-H) cells were shown. I-J, Th1, Th2, Th17, and Treg cells were isolated from the peripheral blood of C57BL/6 mice, labeled with CFST, then exposed to conditioned medium from HT22 cells that had undergone OGD/R with or without TSN treatment. Then CFSE negative cells were evaluated by flow cytometry. Representative histogram (I) and percentage of CFSE negative cells (J) were shown. **P*<0.05.


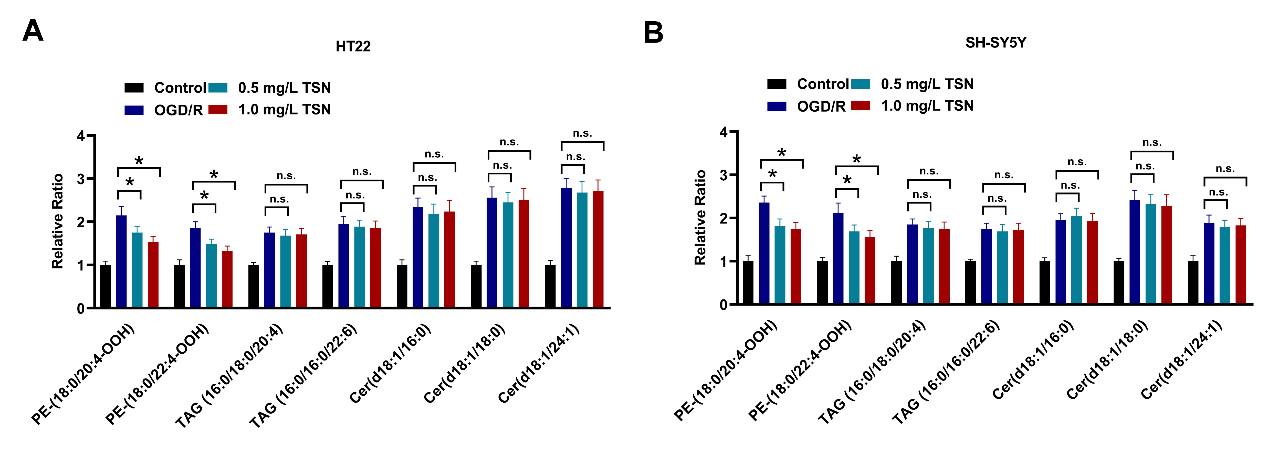


Figure S6. TSN promotes lipid metabolism remodeling *in vitro* after OGD/R. A-B, the contents of indicated lipid species were evaluated by LC-MS. **P*<0.05, n.s.= not significant.


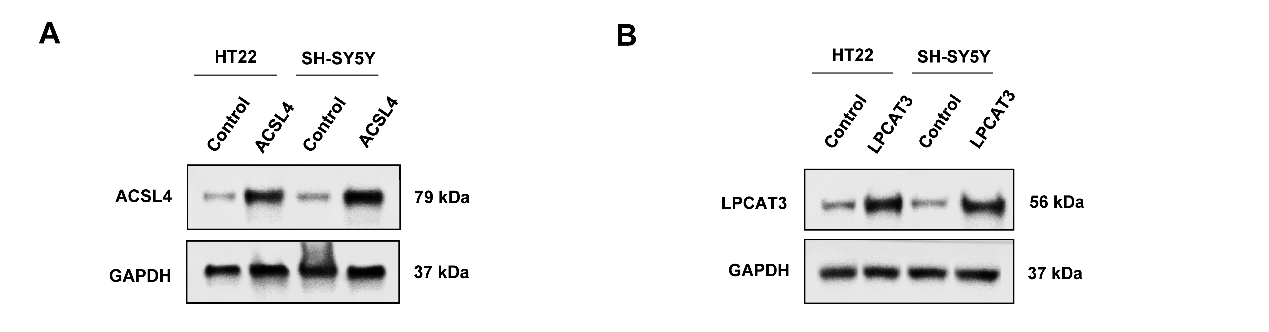


Figure S7. Ectopic overexpression of ACSL4 and LPCAT3 in HT22 and SH-SY5Y cells. HT22 and SH-SY5Y cells were introduced with ACSL4 (A) or LPCAT3 (B) expression vector, then collected lysates for western blot. Empty PCDH vector was used as a negative control.


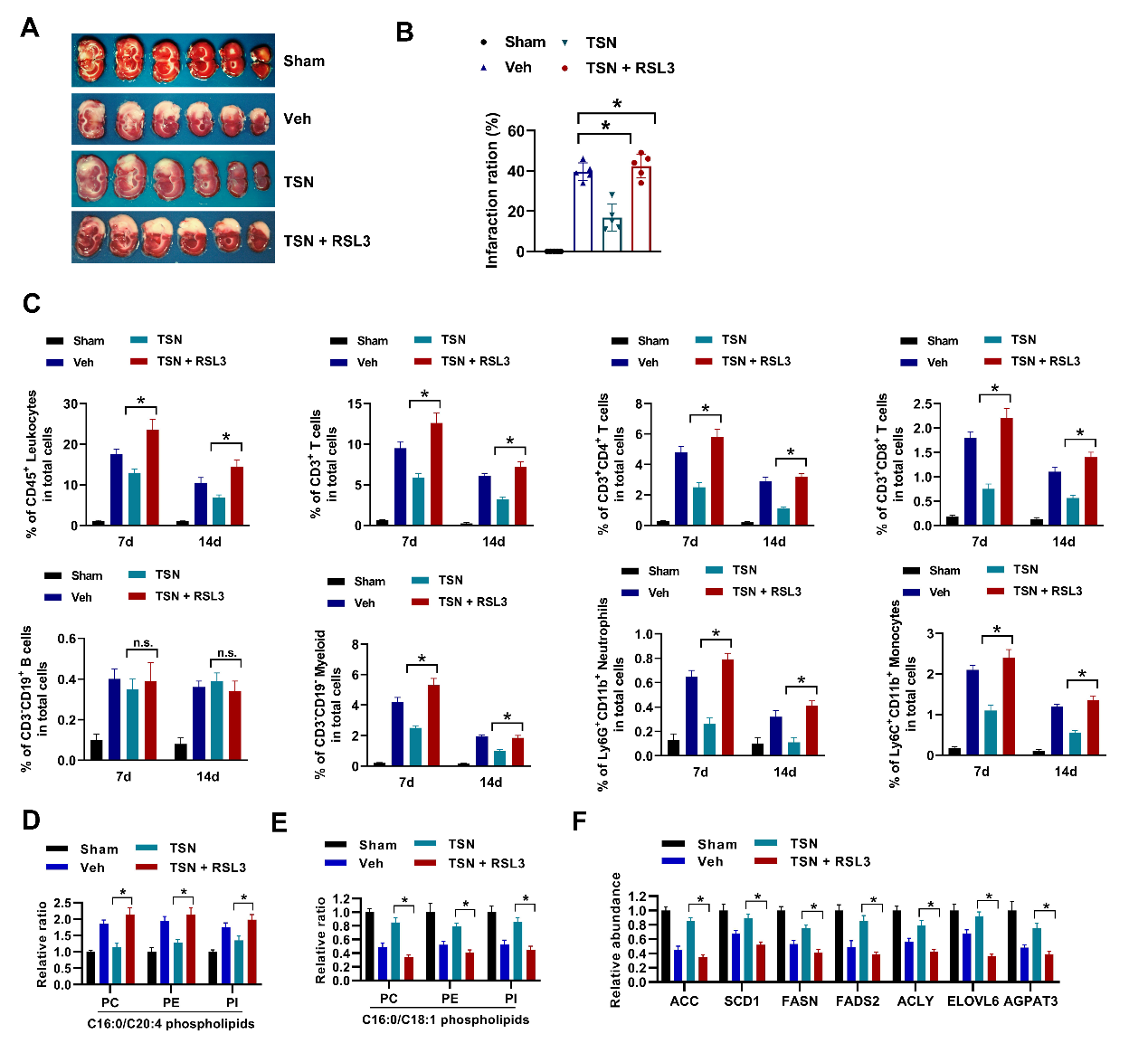


Figure S8. Inducing of ferroptosis abrogated the influence of TSN on immune infiltration and lipid metabolism in MCAO mice. A-B, MCAO mice were treated with 0.5 mg/kg TSN, 3 mg/kg RSL3 or equal volume of Vehicle (Veh) control daily for 14 days, then infarct volume was evaluated by TT staining. Representative images (A) and infarct volumes (B) were displayed. C, infiltrating immune cells in brain tissues were evaluated by flow cytometry. D-F, the contents of C16:0/C20:4 PL-PUFA (D) and C16:0/C18:1 PL-MUFA (E) in PC, PE, and PI of brain tissues of MCAO mice were evaluated by LC-MS. The levels of indicated genes were evaluated by RT-qPCR (F). **P*<0.05.

Supplementary Table 1. Sequences of primers in RT-qPCR.

| Gene | Sense primer | Antisense primer |
| --- | --- | --- |
| Mouse ACC | CAAGACCACCAACGCTAA | GCTGATAGGAAGATAGACTC |
| Mouse SCD1 | CTGGCTGGAGAGTCATCA | ACGAGGACGACAATACAAT |
| Mouse FASN | AGGCTCTCAAGAAGGTGAT | CATTGTACTCGGCAGAAG |
| Mouse FADS2 | GCTTCGTTCTGAGTGTC | GGTCTGGAGTTGTGATGG |
| Mouse ACLY | GTGGTCTGCTGGTGTATC | CCTGGTTCTTGGCTACTG |
| Mouse ELOVL6 | GGCTTGTATGTTCGCTAC | GTTGTCAGTGTGGTGTT |
| Mouse AGPAT3 | CCAACTACGAGCCATCT | GCCTGTTACCTTCCGAT |
| Mouse GAPDH | TCTCCTGCGACTTCAAC | TGTAGCCGTATTCATTGTC |
| Human ACC | CTGCGAGTAGAGACACAAT | GATTCTTCTTGGTGACTTGAG |
| Human SCD1 | CACATCAACTTCACCACATT | AACTCAGCCACTCTTGTAG |
| Human FASN | GCCGCCATCTACAACATC | TTCCACACTATGCTCAGGTA |
| Human FADS2 | TTCTTCAACGACTGGTTCA | CGGCTTCTCCTGGTATTC |
| Human ACLY | CAGCGATACCATCTGTGAT | TAGTCTTGGCATAGTCATAGG |
| Human ELOVL6 | ATCTTCTGGTCCTCACTCA | ATGGCTTCCTCCTCAGTT |
| Human AGPAT3 | CAACAATGTAAGCAGCACTT | CAATACCGCAACGAACAG |
| Human GAPDH | TATGACAACAGCCTCAAGAT | AGTCCTTCCACGATACCA |

**Supplementary Methods**

**Neurological score**

Neurological dysfunction was assessed using the modified neurological severity score (mNSS) by three independent investigators as previously described [1]. The mNSS comprises motor tests (0-6), beam‑balance tests (0-6), and reflexes/absent or abnormal movements (0-2). Total scores of 1-4, 5-9, and 10-14 indicate mild, moderate, and severe impairment, respectively.

**TTC (2,3,5‑triphenyltetrazolium chloride) staining**

Infarct volume was assessed by TTC staining as previously described [1]. Mouse brains were immersion-fixed in optimal cutting temperature (OTC) compound and stored at -20°C, then sectioned coronally at 2 mm thickness, stained with 2% TTC in the dark at 37°C for 20 min, washed twice with PBS, and fixed overnight in 4% paraformaldehyde at 4°C. Infarct volume was calculated as: (contralateral hemisphere − ipsilateral non-infarct area) / contralateral hemisphere × 100%.

**Open-field test and Morris water maze test**

Spatial behavior was assessed using the open‑field test in a 50 × 50 × 30 cm cubic arena. Mice were placed in the center and allowed 10 min of free exploration while movement trajectories, total distance, and mean velocity were recorded. Spatial learning and memory were evaluated in the Morris water maze using a 1.2 m circular tank filled with opaque water at ~22°C. A hidden platform (1 cm below the surface) was positioned in the target quadrant. Mice underwent four trials/day for 6 consecutive days of hidden‑platform pretraining. Each trial was lasted 60 s and mice that failed to find the platform within 1 min were guided to it and allowed 30 s of rest. On day 7, a 60 s probe trial with the platform removed was performed, followed by the formal test in which mice searched for 60 s and data were collected for swimming trajectory, latency to find the platform, number of platform crossings, and time in the target quadrant.

**Hanging wire test, rotarod test and foot-fault test**

Motor function was assessed using the hanging wire test, rotarod test, and foot‑fault test. In the hanging wire test, mice were placed on the lid of a wire‑housed cage. The lid was rapidly inverted, and the time to fall was recorded. In the rotarod test, mice were placed on an accelerating rotating rod (from 0 to 300 rpm over 5 min), and the latency to fall was recorded across three trials. In the foot‑fault test, mice were placed on a stainless‑steel grid floor, and the total number of foot steps and the number of forelimb foot‑faults were recorded.

**Hematoxylin and eosin (H&E) staining**

After intraperitoneal injection of 1% pentobarbital sodium (50 mg/kg), mice were transcardially perfused with 4% paraformaldehyde for 10 min. Then, mice were sacrificed using the neck dissociation method. Decapitated the mice with sharp scissors and made a midline skin incision to expose the skull. The skull was then opened by cutting along the midline suture from the foramen magnum rostrally toward the eyes using fine scissors. The skull bones were gently retracted laterally with fine forceps to expose the brain. The brain was subsequently lifted from the olfactory bulbs, and the cranial nerves at its base were severed with a spatula or fine forceps. Immediately upon removal, the brain tissues were overnight fixed by 4% paraformaldehyde at 4°C. Next, brain tissues were embedded in paraffin, sectioned at 8 μm, and stained with H&E (Beyotime, China) following the manufacturer’s protocol. The denatured cell index was calculated as degenerated cells/total cells, and images were acquired using a Nikon microscope (Japan).

**Terminal deoxynucleoitidyl transferase dUTP nick-end labeling (TUNEL) staining**

Apoptotic cells were detected via TUNEL staining (Beyotime, China) following the manufacturer’s protocol. Brain sections were fixed in 4% paraformaldehyde at 4°C overnight, incubated with the TUNEL mixture at 37°C for 30 min, washed with PBS, and visualized using DAB reagents. Images were captured with a Nikon microscope (Japan).

**Enzyme-linked immunosorbent assay (ELISA)**

Levels of IL‑1β, TNF‑α, IL‑6, IL‑10, TGF‑β and 4-HNE in brain tissues, serum or cell samples were quantified using ELISA kits (Abcam, USA) following the manufacturer’s protocol. Briefly, samples were incubated in antibody‑coated plates for 1 h at room temperature, washed five times, then sequentially incubated with biotinylated antibodies, streptavidin antibodies, substrate solution, and stop solution. All samples were run in triplicate, and absorbance at 450 nm was measured with a microplate reader.

**Flow cytometry**

Infiltrating immune cells were analyzed by dissociating brain tissues into single-cell suspensions using collagenase (1 mg/mL) and DNase (10 mg/mL) and filtering through a 70 μm strainer. To disassociate culture cells into single cell suspension, adherent cells were digesting with 0.25% trypsin, then filtered through a 70 μm strainer. 1×10^6^ cells were blocked with anti-FC receptor antibody, stained with flow cytometry antibodies (4°C, 30 min). Intracellular staining were performed using the ThermoFisher kit (USA) per manufacturer’s instructions. Cell proliferation was assessed by staining with 1.25 μM CFSE for 10 min. Flow cytometry was performed on a CytoFLEX (Beckman Coulter, USA) with data analyzing via FlowJo (Treestar, USA). Flow cytometry antibodies used in our study were CD3 APC, CD4 PE, CD8 PE, CD19 PE, CD25 PerCP-Cy5.5, FoxP3 PE-Cy7, CD11b APC, Ly6G PE, Ly6C FITC, CD45 PE-Cy7, IL-17A APC-Cy7, IFN-γ FITC, C11 BODIPY and IL-4 FITC (all from eBioscience, USA). The gating strategy of flow cytometry analysis was shown in Supplementary Figure 1.

***In vitro* T cell activation, differentiation and co-culture experiments**

To collected peripheral blood of C57BL/6 mouse from the facial vein, mice were anesthetized with 1% pentobarbital sodium (50 mg/kg) intraperitoneally, then a sterile lancet is used to puncture the vessel at a defined landmark on the cheek, allowing blood to drip directly into a collection tube. Peripheral blood (0.2 mL) was collected from each mouse and the blood collection was a non-terminal procedure. Murine CD4+ T cells were isolated from peripheral blood of C57BL/6 mouse using Stemcell CD4 T cell isolation kits (Canada), then cultured in DMEM medium supplemented with 10% fetal bovine serum. For in vitro activation, 1×10^6^ naïve CD4+ T cells were seeded into 6‑well plates and stimulated for 72 h with anti‑CD3 (0.25 μg/mL, Biolegend #100340, USA) and anti‑CD28 (0.50 μg/mL, Biolegend #102116, USA). For Treg differentiation, naïve CD4+ T cells were cultured with anti‑IL‑4 (2 μg/mL, Biolegend #504122, USA), anti‑IFN‑γ (2 μg/mL, Biolegend #517906, USA), IL‑2 (20 ng/mL, Biolegend #575404, USA), and TGF‑β (1 ng/mL, Miltenyi Biotech #130‑095‑066, USA). For Th17 differentiation, naïve CD4+ T cells were cultured with anti‑IL‑4 (2 μg/mL, Biolegend #504122, USA), anti‑IFN‑γ (2 μg/mL, Biolegend #517906, USA), IL‑6 (30 ng/mL, Biolegend #575704, USA), and TGF‑β (0.3 ng/mL, Miltenyi Biotech #130‑095‑066, USA). For Th1 differentiation, naïve CD4^+^ T cells were cultured with anti‑IL‑4 (2 μg/mL, Biolegend #504122, USA) and IL‑12 (20 ng/mL, Biolegend #577004, USA). For Th2 differentiation, naïve CD4+ T cells were culture with anti-IFN-γ (10 μg/mL, Biolegend #517906, USA) and IL-4 (20 ng/mL, Biolegend #574304, USA). For co-culture experiments, 1×106 naïve T cells isolated from mouse PBMCs were co-cultured via a transwell system (Falcon, #FAL-353097, 8 μm pore size) with 1×106 HT22 cells that had been pre-exposed to OGD/R with or without TSN treatment (0.5 mg/L or 1 mg/L), then exposed to Th1, Th2, Th17 or Treg polarizing conditions for 72 h as described above.

**Cell culture**

The mouse hippocampal neuronal cell line HT22 was obtained from the Institute of Biochemistry and Cell Biology (Chinese Academy of Sciences, Shanghai) and the human neuroblastoma cell line SH-SY5Y was obtained from American Type Culture Collection (ATCC, # CRL-2266). Cells were routinely cultured in DMEM (Gibco, USA) supplemented with 10% heat-inactivated FBS (Gibco, USA) at 37°C in 5% CO₂.

**Plasmid constructs**

ACSL4 and LPCAT3 overexpression were conducted by inserting the coding sequences of mouse ACSL4 (Accession number: NM_207625.2), human ACSL4 (Accession number: NM_001318509.2), mouse LPCAT3 (Accession number: NM_145130.3) or human LPCAT3 (Accession number: NM_005768.6) into the PCDH vector (System Biosciences #CD510B, USA). Empty PCDH vector was used as a negative control. ACSL4 Sh#1 and Sh#2 targeting both mouse and human ACSL4 was generated by cloning the specific short hairpin RNA sequences into the PLKO.1 vector. PLKO.1 vector inserted with non-targeting short hairpin RNA was used as Scramble control (Scr: 5’-ACGGA GGCTA AGCGT CGCAA-3’). The ACSL4 Sh#1 and Sh#2 sequences were: Sh#1, 5’-TCCAC TTGTT AATAA CAATA TAC-3’, Sh#2, 5’-GCCAT GAAAT TGGAG CGATTT-3’.

**Cell viability assay**

Cell viability was assessed using the Cell Counting Kit-8 (CCK-8, Takara, Japan) following the manufacturer's protocol. HT22 and SH-SY5Y cells (2,500 cells/well) were seeded in 96-well plates and treated as indicated, then incubated with 10 μL CCK-8 reagent for 1 h at 37°C. Absorbance at 450 nm was measured spectrophotometrically, with all samples tested in triplicate.

**Western blot**

Cellular lysates and tissue homogenates were prepared using ice-cold RIPA lysis buffer (Beyotime, China) with protease inhibitors (Sigma, USA), followed by BCA protein quantification (Beyotime, China). Proteins were separated on 8-12% SDS-PAGE gels and transferred to PVDF membranes (Millipore, USA), then incubated overnight at 4°C with primary antibodies and 1 h at RT with secondary antibodies. Protein bands were visualized using an ECL kit (ThermoFisher, USA). Western blot antibodies included: HO-1 (Cell Signaling #43966, 1:1000), Transferrin (Invitrogen #PA5-27306, 1:1000), GPX4 (Cell Signaling #52455, 1:1000), SLC7A11 (Abcam #ab307601, 1:500), GAPDH (Cell Signaling #2118, 1:1000), ACC (Cell Signaling #3676, 1:1000), FASN (Cell Signaling #3180, 1:1000), FADS2 (Abcam #ab314317, 1:500), SCD1 (Cell Signaling #2794, 1:1000), ACSL4 (Abcam #EPR8640, 1:500), β-Tubulin (Cell Signaling #2146, 1:1000) and LPCAT3 (Cell Signaling #72964, 1:1000). All samples were tested in triplicate.

**Measurement of reactive oxidative species (ROS), (Malondialdehyde) MDA and (Glutathione) GSH**

Cellular and mitochondrial ROS were measured using DCF-DA and MitoSox Red probes, respectively. HT22 and SH-SY5Y cells were seeded in 6-well plates, incubated with 10 μM DCF-DA or 5 μM MitoSox Red at 37°C for 30 min in the dark, then analyzed by flow cytometry. MDA and GSH levels were quantified using Nanjing Jiancheng kits (#A003-4-1 for MDA, #A006-2-1 for GSH) following the manufacturer's protocols, with all samples tested in triplicate.

**Measurement of iron levels**

Intracellular chelatable iron was measured using Phen Green SK probe (Thermo Fisher Scientific #P-14313, USA) by incubating cells with 5 μM probe for 30 min at 37°C, followed by PBS washing and 4% PFA fixation for 10 min at RT. Fluorescence imaging was performed with a Leica TCS SP8 confocal microscope (Switzerland). Cellular ferrous iron was quantified via Sigma iron assay kit (MAK025, USA). Cells were homogenized in assay buffer, centrifuged at 13,000 rpm for 10 min at 4°C. and 100 μL supernatant was mixed with 5 μL assay buffer for 30 min at RT, then incubated with 100 μL iron probe for 60 min at RT avoiding light. Absorbance at 593 nm was measured spectrophotometrically, with all samples tested in triplicate

**Drug affinity–responsive target stability (DARTS)**

Cells were lysed, and total proteins were separated using lysis buffer, followed by centrifugation at 12,000g for 10 min at 4°C. The supernatant was treated with DMSO or TSN, incubated at 4°C for 2 h, then digested with pronase (Roche, #10165921001) at RT for another 2 h. The reaction was stopped by adding protease inhibitors and SDS-PAGE sample buffer prior to Western blotting.

**Cell thermal shift assay (CTSA)**

Cells were lysed in NP-40 buffer containing phosphatase inhibitors for 30 min at 4°C, followed by centrifugation at 12,000g for 10 min at 4°C. The protein supernatant was treated with DMSO or TSN and incubated at 4°C for 2 h, then heated at 37-58°C for 5 min and recentrifuged at 12,000g for 10 min at 4°C. Supernatants were analyzed via Western blotting.

**Recombinant ACSL4 protein purification and ACSL4 activity assay**

To achieve the production of the ACSL4 protein, mouse ACSL4 (Accession number: NM_207625.2) was cloned into the pGEX-6P-1 vector (GE Healthcare, USA), then the recombinant plasmid harboring the corresponding gene was first introduced into E. coli BL21 (DE3) cells. Following transformation, the expression of the recombinant protein was induced by adding 0.1 mM IPTG, and the culture was incubated at a low temperature of 16°C for an extended period of 24 hours to facilitate proper folding and soluble expression. Subsequently, the induced cells were harvested, and the target protein was affinity-purified using glutathione S-transferase (GST)-tag specific resins. The bound protein was eluted with a buffer containing 20 mM Tris-HCl (pH 8.0), 150 mM NaCl, and 1 mM dithiothreitol (DTT). Finally, the eluted protein was aliquoted and stored at -80°C to preserve its stability for future experiments. The ACSL4 activity was assessed using a previously described method by co-incubating 100 ng recombinant ACSL4 protein with varying TSN concentrations for 10 min at 37°C in a 100 μL reaction mixture (175 mM Tris-HCl pH 7.4, 10 mM ATP, 8 mM MgCl₂, 5 mM DTT, 250 μM CoA, 50 μM [³H]-AA) [2]. After reaction termination with 1 mL ethyl acetate, the organic layer was discarded, and the aqueous phase was extracted twice. Radioactivity was measured by liquid scintillation counting, with results expressed as percentage of total enzymatic activity.

**References**

[1] X. Li, Q. Xia, M. Mao, H. Zhou, L. Zheng, Y. Wang, Z. Zeng, L. Yan, Y. Zhao, J. Shi, Annexin-A1 SUMOylation regulates microglial polarization after cerebral ischemia by modulating IKKalpha stability via selective autophagy, Sci Adv 7(4) (2021) eabc5539.

[2] Z. Zhang, Z. Yao, L. Wang, H. Ding, J. Shao, A. Chen, F. Zhang, S. Zheng, Activation of ferritinophagy is required for the RNA-binding protein ELAVL1/HuR to regulate ferroptosis in hepatic stellate cells, Autophagy 14(12) (2018) 2083-2103.
